# Transcriptomic analysis identifies a tumor subtype mRNA classifier for invasive non-functioning pituitary neuroendocrine tumor diagnostics

**DOI:** 10.7150/thno.47525

**Published:** 2021-01-01

**Authors:** Xinjie Bao, Gengchao Wang, Shan Yu, Jian Sun, Liu He, Hualu Zhao, Yanni Ma, Fang Wang, Xiaoshuang Wang, Renzhi Wang, Jia Yu

**Affiliations:** 1 Department of Neurosurgery, Pituitary Center, Peking Union Medical College Hospital (PUMCH), Chinese Academy of Medical Sciences (CAMS) & Peking Union Medical College (PUMC), Beijing 100730, PR China.; 2 State Key Laboratory of Medical Molecular Biology, Institute of Basic Medical Sciences, Chinese Academy of Medical Sciences (CAMS) & School of Basic Medicine, Peking Union Medical College (PUMC), Beijing 100005, PR China.; 3 Department of Biochemistry, Institute of Basic Medical Sciences, Chinese Academy of Medical Sciences (CAMS) & School of Basic Medicine, Peking Union Medical College (PUMC), Beijing 100005, PR China.; 4 Department of Pathology, Peking Union Medical College Hospital (PUMCH), Chinese Academy of Medical Sciences (CAMS) & Peking Union Medical College (PUMC), Beijing 100730, PR China.; 5 Medical Epigenetic Research Center, Chinese Academy of Medical Sciences (CAMS), Beijing 100005, PR China.

**Keywords:** Pituitary neuroendocrine tumors, transcriptome, diagnostic panel, INSM1, HSPA2

## Abstract

**Rationale:** The invasive behavior of non-functioning pituitary neuroendocrine tumors (NF-PitNEts) presents obstacles for complete surgical resection and is indicative of poor prognosis. Therefore, developing reliable diagnostic tools for identifying invasive PitNEts would be helpful in guiding surgical decisions and, in particular, the follow-up treatment.

**Methods:** We analyzed differential gene expression profiles between 39 non-invasive and 22 invasive NF-PitNEts by high-throughput sequencing, gene co-expression, and functional annotation. Twenty-one transcripts were further validated by Taqman-qPCR in another 143 NF-PitNEt samples. The histological expression and serum-exosomal mRNA of three candidate genes were examined by tissue microarray and droplet digital PCR.

**Results:** Non-invasive and invasive NF-PitNEts were clustered into distinct groups with a few outliers because of their gonadotroph, corticotroph, or null cell lineages. The gene signature with strong invasive potential was enriched in 'Pathways in cancers' and 'MAPK pathway', with significantly higher *in situ* INSM1 and HSPA2 protein expression in invasive NF-PitNEts. Further integration of the 20 qPCR-validated differentially expressed genes and pituitary cell lineages provided a gene-subtype panel that performed 80.00-90.24% diagnostic accuracy for the invasiveness of NF-PitNEts.

**Conclusion:** Our approach defined new characteristics in the core molecular network for patients at risk for invasive NF-PitNEt, representing a significant clinical advance in invasive PitNEt diagnostics.

## Introduction

Pituitary neuroendocrine tumors (PitNEts) are common intracranial and neuroendocrine tumors that arise mostly in a sporadic manner and only a minority of adenomas is part of hereditary or familial syndromes 1, 2. Non-functioning pituitary adenomas (NF-PitNEts), with signs and symptoms of local mass effect or hypopituitarism, represent the second most frequent pituitary tumor (~30%-40%) after prolactinomas 3. All NF-PitNEt patients present without an endocrine hypersecretion syndrome but display heterogeneity in morphology and biologic features 3, 4. As per the World Health Organization (WHO) classification of endocrine tumors, clinically, NF-PitNEts are divided into null cell adenomas that exhibit negative immunoreactivity for both pituitary hormones and pituitary transcription factors, and silent adenomas that show histological and immunohistochemical features consistent with a well-differentiated, lineage-specific adenoma 4, 5. Null cell adenomas tend to be macroadenomas with tumor cells arranged in a variety of histopathological patterns 4, while most gonadotroph adenomas, as well as up to 20% of corticotroph adenomas, constitute the most commonly encountered clinically silent tumors ( SGA and SCA) 6.

Although mostly considered benign, 35% of PitNEts at the time of surgery are invasive NF-PitNEts that can aggressively invade surrounding structures 7. According to the grading system developed by Knosp 8, the increasing grade of PitNEts correlates with the cavernous sinus invasion, especially in those classified as grade 3 or 4. Invasive adenomas are generally macroadenomas that are larger than 10 mm 9. Interestingly, some patients with macroadenomas and even giant adenomas do not present distinctive histopathological features of invasion and/or aggressiveness, and this phenomenon is more common for NF-PitNEts 10, 11.Certain types of PitNEts, such as SCA, are specially recognized as “high-risk PitNEts” due to their clinically aggressive behavior 12. Therefore, tumor invasion is correlated with cell lineage, radioanatomical characteristics, and ultrastructural features.

Currently, invasive PitNEts are challenging to manage due to incomplete surgical resection requiring chemotherapy and/or radiotherapy. However, both chemotherapy and radiotherapy remain difficult for some invasive PitNEts, which tend to recur quickly and have a fatal outcome 13, 14. Indeed, anatomical invasion is considered a critical prerequisite for malignant prognosis by the new WHO classification system 4. Identifying accurate features indicative of invasive NF-PitNEts will guide surgical decision-making and particularly the use of follow-up treatment, preventing recurrence at early stages.

Previous efforts have identified *in situ* molecular markers, including Ki-67, p53, and p27, for invasive PitNEts, 8, 15, 16. However, their levels and immunohistochemical (IHC) staining tend to vary depending on the evaluation method and could change when PitNEts are further classified into subgroups 17. Since classic oncogene mutations are rarely encountered in NF-PitNEts and somatic genetic alterations are also infrequent 5, 18, 19, identification of the transcriptomic signatures underlying the invasiveness of NF-PitNEts is crucial. A few studies using meta-analysis or omics-driven data approaches proposed mRNA or microRNA markers for invasive NF-PitNEts 20-23. Given the inadequate data for the precise characterization of NF-PitNETs, it is a clinically meaningful endeavor to develop reliable diagnostic tools for tumor invasion detection for advancing the management of this disease.

In this study, we characterized mRNA profiles of noninvasive and invasive NF-PitNEts and identified distinct molecular features among different tumor subtypes. We searched for transcriptomic signatures and signaling pathways that play important roles in the invasive NF-PitNEts and investigated their potential for differentiating these tumors.

## Methods

### Sample collection (clinical diagnosis) and study oversight

Informed consent was obtained from all individual participants included in this study. The study recruitment processes and protocol were approved by the Ethics Review Committee of Peking Union Medical College Hospital (No. S-551). PitNEts were obtained from patients who underwent transsphenoidal surgery at Peking Union Medical College Hospital between May 2012 and July 2017. There were 61 NF-PitNEts for RNA-seq, 143 NF-PitNEts for TaqMan quantitative RT-PCR and tissue microarray. The diagnosis of NF-PitNEts was based on clinical manifestation, hormonal test and magnetic resonance imaging (MRI) examination. Additionally, immunohistochemical staining for all anterior pituitary hormones and transcription factors was performed to classify the subtypes of NF-PitNEts (Table S1). In this study, maximum diameter of all NF-PitNEts was greater than 20 mm. Non-invasive PitNEts and invasive PitNEts were diagnosed according to Knosp classification and were intraoperatively confirmed 24. Non-invasive PitNEts were Knosp grade 0, 1, or 2, and importantly did not invade the cavernous sinus. All invasive PitNEts included in this study were Knosp grade 4 and invaded the cavernous sinus definitely. The clinical characteristics, operative findings, postoperative complications, pathological results, and follow-up data were recorded for each included patient.

### RNA extraction and sequencing

Total RNA was extracted from the tissues of PitNEts, and RNA integrity was examined using a NanoDrop 2000/2000c (Thermo Fisher Scientific Inc., Waltham, MA, USA). The RNA library was constructed using TruSeq® RNA LT Sample Prep kit v2 (Illumina Inc., San Diego, CA, USA) according to the manufacturer's instructions. Briefly, after poly (A)-based mRNA enrichment and RNA fragmentation, first-strand complementary DNA (cDNA) was synthesized using the First Strand Master Mix and SuperScript II, followed by second-strand cDNA synthesis using the Second Strand Master Mix. Double-stranded cDNA was end repaired, and then 3' ends were adenylated and the illumina adaptors were ligated. The ligation product was amplified with 15 cycles of polymerase chain reaction. After measures of yield and fragment length distribution, libraries were sequenced using TrusSeq SBS kit v3-HS, and were generated on the HiSeq 2500 or 3000 sequencing system (both from Illumina Inc.). Raw reads were trimmed the adaptor sequences with Trim Galore. Sequencing reads were mapped to human genome GRCh38 with Gencode 25 v28 annotations using TopHat2. Gene expression levels were quantified with Cufflinks 26 and HTSeq 27.

### Exosome extraction from peripheral blood

The peripheral blood of patients was drawn into Vacuette® tubes with serum separator clot activator (GREINER BIO-ONE, Germany). The blood samples were placed at room temperature for 10 min, centrifuged at 1900 × g, 4°C for 10 min. The supernatant was transferred into new conical-bottom centrifuge tubes, then centrifuged at 1600 × g for 10 min at 4°C to remove cells and debris. After centrifugation, the supernatant containing clarified serum was carefully transferred into a new tube without disturbing the pellet. Exosomes were next extracted from clarified serum using Total Exosome Isolation Reagent (from serum) (Thermo Fisher Scientific Inc., Waltham, MA, USA) according to the manufacturer's instructions. The pelleted exosomes were resuspended in PBS for transmission electron microscopy (TEM) and nanoparticle tracking analysis (NTA) by the NanoSight NS300 instrument.

### Exosomal RNA purification and reverse transcription

For total RNA extraction, the exosome pellet was lysed with 1mL QIAzol Lysis Reagent, and proceeded using miRNeasy Micro Kit (QIAGEN, Germany). Then aliquots (11 μL) from total RNA were reverse transcribed using SuperScript™ III Reverse Transcriptase (Thermo Fisher Scientific Inc., Waltham, MA, USA) following the manufacturer's instructions.

### Droplet digital PCR (ddPCR)

The ddPCR reaction was performed following the recommendations of the supplier (Bio-Rad, California, USA). The reaction system is as follows: 1 × ddPCR™ Supermix for Probes (no dUTP) (Bio-Rad), 800 nM primer, 250 nM probe and 3 μL of cDNA of template, ddH_2_O up to 20 μL. After homogenization, the ddPCR reaction mixture and 70 μL of oil (Bio-Rad) were respectively loaded into the corresponding wells of droplet generator cartridge (Bio-Rad). The ddPCR reaction mixture were then produced an emulsion about 40 μL by QX200™ Droplet Generator, subsequently transferred to a 96-wells PCR plate. The plate was heat-sealed by a pierceable foil (Bio-Rad) and subjected to thermal cycling conditions: 95°C for 5 min for Taq polymerase activation; 39 cycles of 94°C for 30 s, 60°C for 40 s; then 98°C for 10 min and held at 12°C. Following PCR, the fluorescence was read on a QX200 droplet reader. Analysis of the result was performed with QuantaSoft software (version 1.7.4.0917). The sequences of primers and probes were listed in Table S2. Expression of GAPDH was used as internal control as previously reported 28.

### Unsupervised clustering and principal component analysis

Unsupervised hierarchical clustering analysis and PCA were performed with top 5000 highest variance expression levels of genes. Expression levels were normalized via log transformation implemented by DESeq2 29. Average was used as the agglomeration method in clustering. The expression levels were scaled among samples when drawing clustered heatmaps.

### Differential gene expression

Differential gene expression (DGE) analysis was conducted using DESeq2. The likelihood ratio test on the difference in deviance between a full and reduced model formula was used to evaluate the significance of interactions. Technical covariates and batch effects were checked before DGE analysis. Significant results were reported at FDR < 0.05. The replication between datasets were evaluated by comparing the squared correlation (R^2^) of log2 fold change of genes in each dataset.

### Weighted gene co-expression network analysis

Weighted gene co-expression network analysis (WGCNA) was performed using the R package WGCNA 30. Signed co-expression network was constructed with normalized counts via the function rlog in DESeq2. The biweight mid-correlation was used as the correlation method in all function in WGCNA. Soft power 17 was set for network construction and module detection. To reduce the number of modules, highly correlated modules with a threshold of 0.8 were merged.

In module-trait analysis, the eigengene of each module was related to the traits of nonfunctioning pituitary adenomas patients. Significant module-trait results were reported at *P* < 0.05.

### Functional annotation

GO and KEGG enrichment analysis were conducted by DAVID 31 using DGEs and genes from significant modules. The Benjamini-Hochberg method was used to correct the multiple comparisons. Gene set enrichment analysis (GSEA) were run on pre-ranked gene lists using the software GSEA 32. The method that was used to generate the ranked lists is as follows:

(1): 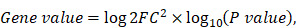 if log2*FC* > 0;

(2): 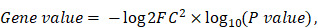
if log2*FC* < 0

The enrichment statistic was set to classic as a more conservative scoring approach.

### Support vector machine classifier

We used the support vector machine (SVM) algorithm implemented by the R package e1071 33 to classify the invasive nonfunctioning pituitary adenomas and non-invasive nonfunctioning pituitary adenomas. The normalized counts of experimentally validated genes were used to construct the SVM algorithm. The SVM algorithm was trained on the training dataset to build the model, and predicted the samples in validation dataset (training:validation = 2:1). Leave-one-out cross validation (LOOCV) on the training dataset was performed to assess the quality of the model. The predictive strength was evaluated by receiver operating curve (ROC) analysis implemented in the R package pROC 34**.**

### Quantitative real-time PCR (qRT-PCR)

mRNA expression was assessed using the TaqMan probe based gene expression analysis (Thermo Fisher, Foster City, CA). The sequences of primers and probes were listed in Table S2, and the measurements were normalized using the UBC and GAPDH 35. The relative expression level in each sample was recorded as the ratio of gene expression to the geometric mean of two reference genes (GAPDH and UBC) expression 36. Three replicates were performed for each sample. The standard curves for GAPDH, UBC, and other mRNAs showed good linearity between Cq values and the log of sample concentrations (Figure S7).

### Tissue microarray (TMA) (including Immunohistochemistry and scoring)

Immunohistochemical studies of the human pituitary adenoma tissue microarrays were done using the following antibodies: INSM1 (sc-271408, dilution 1 : 200; Santa cruz); HSPA2 (HPA000798, dilution 1 : 100; Sigma-Aldrich); CDK6 (H00001021-M01, dilution 1 : 50; Abnova); T-PIT (ZM-0318, dilution 1 : 100; ZSJQ Corp.); SF-1 (ZM-0089, dilution 1 : 100; ZSJQ Corp.); and PIT-1 (ZM-0208, dilution 1 : 100; ZSJQ Corp.), which was stained in serial-cut tissue array sections as previously described 37. TMAs were de-paraffinised in xylene and hydrated in ethanol and rinse under water. Endogenous peroxidase activity was blocked for 30 min with 3% H_2_O_2_. After a 3 h block with EnvisionTM FLEX Peroxidase-Blocking Reagent (Dako#SM801, Agilent, Santa Clara, CA), the primary antibody was used overnight. EnvisionTM FLEX/HRP (Dako#SM802, Agilent) was used as secondary antibody and chromogenic detection was carried out using EnvisionTM FLEX Substrate Buffer+EnvisionTM FLEX DAB+ Chromogen (Dako#SM803, Agilent).

Images were taken with an Axio Scan.Z.1 (ZEISS, Germany). The staining results were assessed by a pathologist specialized in neuropathology and blinded to the patient details using the staining H-score method 38. The score was obtained by computing staining intensity of each cell (0, 1, 2, 3) and the proportion of pituitary cells stained for each intensity (0-100) to give a score between 0 and 300, using the formula: 3 × percentage of strongly staining cell + 2 × percentage of moderately staining cell + 1 × percentage of weakly staining cell. Appropriate tissues were used as positive controls, and negative controls were obtained by substituting the primary antibodies with non-immune rabbit or mouse sera.

### Statistical analysis

Data are shown as means ± S.D. or for maximum value, 75^th^ percentile, 50^th^ percentile, 25^th^ percentile and minimum value. Chi-squared test or fisher's exact test was used to the statistical test of categorical clinical characteristics of pituitary adenoma patients dependent on the theoretical frequency. Mann-Whitney U test or t-test after confirming equality of variance by F test was used for continuous variables.

### Availability of data and materials

The RNA sequencing data generated and analyzed in the current study can be accessed from Genome Sequence Archive (GSA; http://bigd.big.ac.cn/gsa or http://gsa.big.ac.cn) with the accession number CRA001236.

## Results

### Clinical features of NF-PitNEt patients

**Figure 1:**
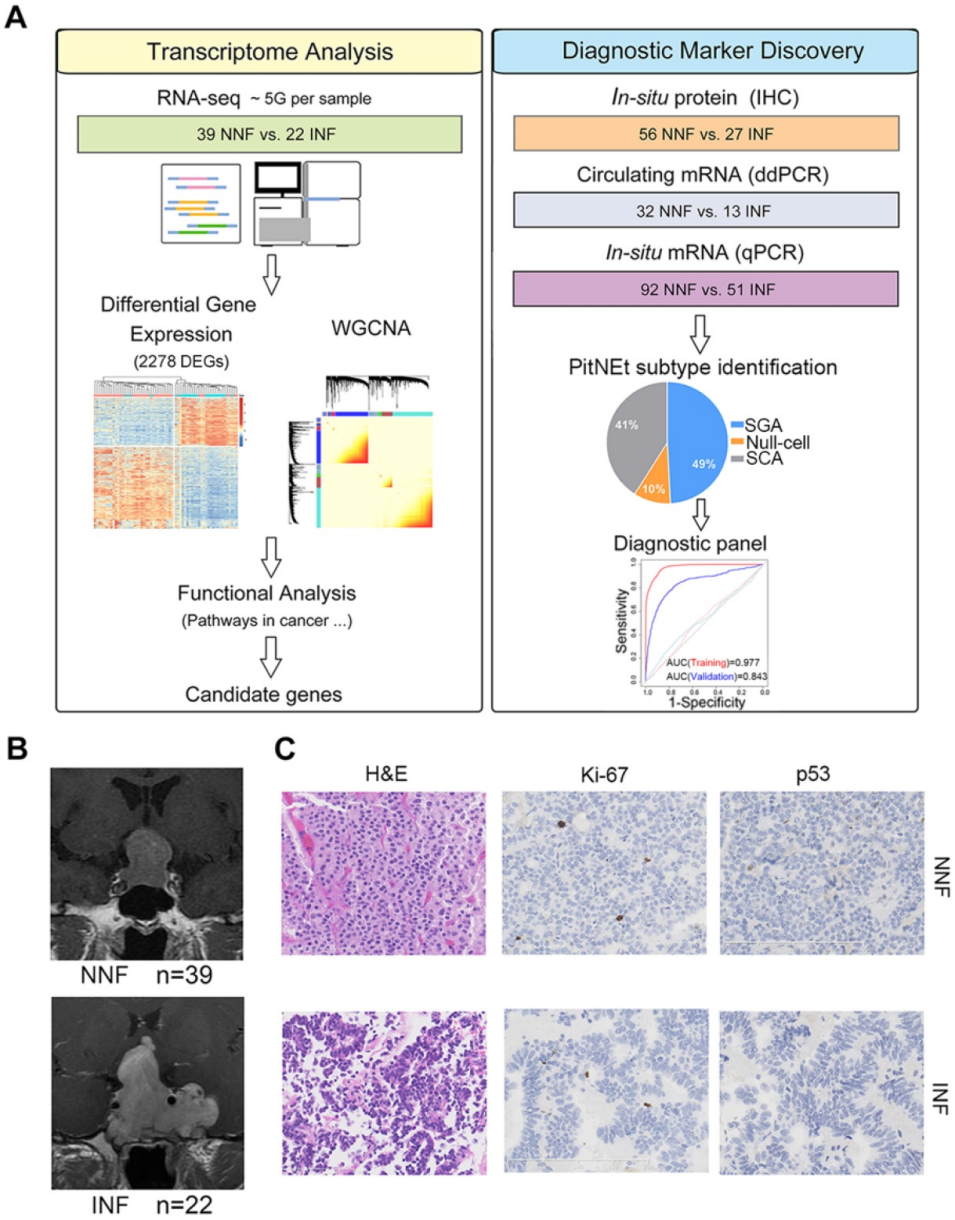
** Identification of the pituitary neuroendocrine tumor samples.** (**A**) Study design scheme. (**B**) Contrast-enhanced, T1-weighted coronal MRI scan of NNF- and INF-PitNEts. Images of INF showing the tumor invading the left cavernous sinus and surrounding the internal carotid artery. Images of NNF showing the tumor did not invade the cavernous sinus. (**C**) H&E staining and immunohistochemical analysis of Ki-67 and p53. Immunostaining for both markers was similar between each group (original magnification, 200×; scale bar represents 100 µm or 200 µm).

To determine the core gene characteristics of tumor invasiveness, we developed a systems biology approach whereby high-throughput transcriptomic analyses and experimental validation were combined (Figure 1A). We obtained clinical NF-PitNEts without any endocrine hypersecretion syndrome and examined the pathological features of tumors. To define the status of invasion into the cavernous sinus, we determined the radiological characteristics by MRI (Figure 1B) with the class criteria described by Knosp *et al.* 8 and confirmed during the operation. A total of 39 tumors with grade 0, 1, or 2 (non-invasive NF, NNF) and 22 tumors with grade 4 (invasive NF, INF) were included in this study. The hematoxylin and eosin (H & E) staining characteristics and the positive rate of p53, and Ki-67 detection were similar between the two groups (Table 1 and Figure 1C).

Surgical results, postoperative complications, and prognosis differed between NNF- and INF-PitNEts. All NNF-PitNEts got grosstotal (74.4%) or subtotal resection (25.6%), whereas more than a half (54.5%) of INF-PitNEts only had a partial resection. Therefore, significantly more INF-PitNEt patients received follow-up treatments, including radiation, medication, and surgery. During follow-up, 14 out of the 22 patients with INF-PitNEt had stable remnants while 8 patients had tumor growth, among which 2 patients died after a mean of 60.4 months. In contrast, there was no tumor growth or death in the NNF group during a mean follow-up of 58.2 months. Furthermore, all INF-PitNEts but only 8.6% NNF-PitNEts suffered postoperative symptoms, such as headache, visual dysfunction, monocular blindness, panhypopituitarism, and cranial nerve palsy (Table 1).

### mRNA profiles show a distinct separation of INF- from NNF-PitNEts

**Figure 2:**
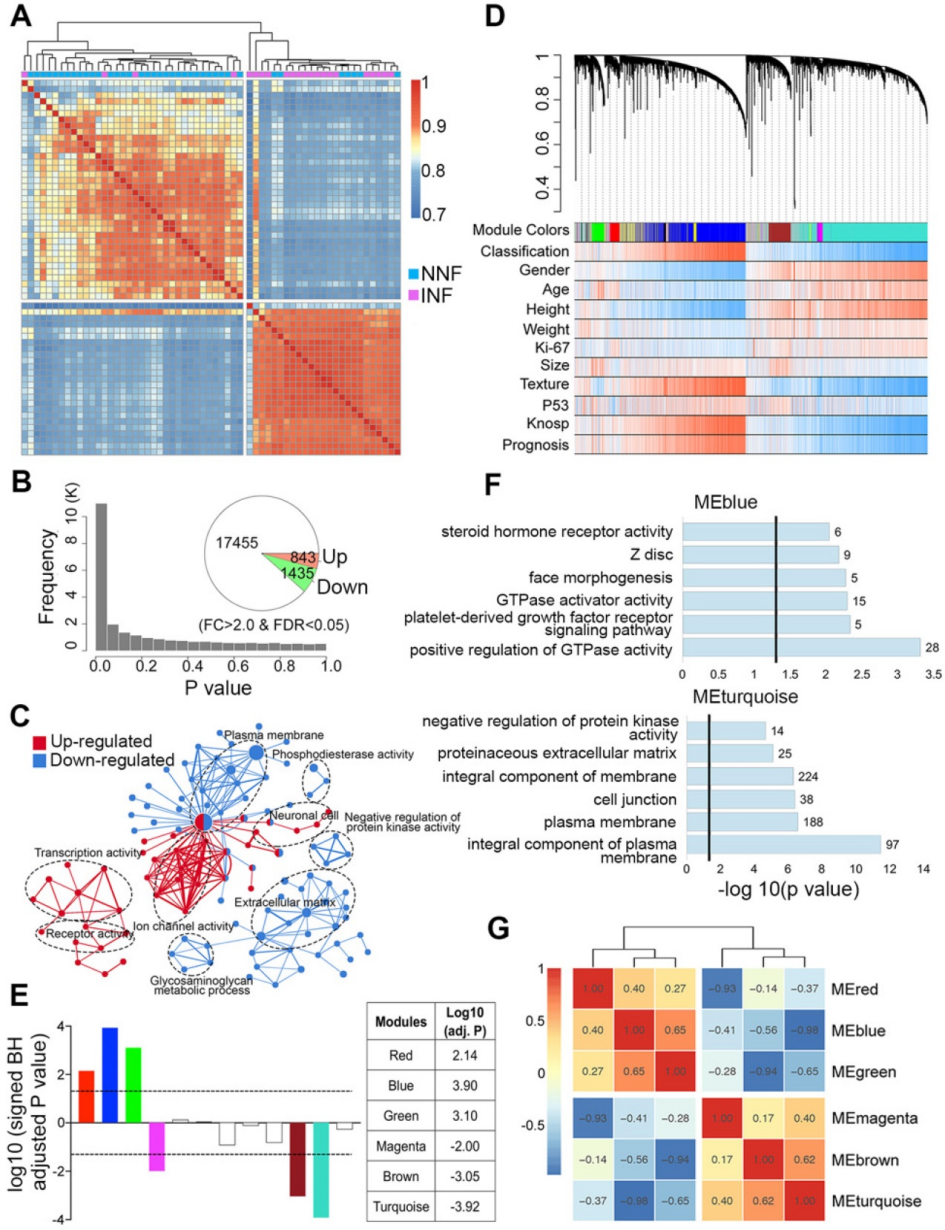
** Differential gene expression and WGCNA of NNF- and INF-PitNEts.** (**A**) Unsupervised hierarchical clustering and heatmap of sample-sample correlations among NF-PitNEts. (**B**) Distribution of *P* values and the number of DEGs between NNFs and INFs. (**C**) GO term enrichment for genes up-regulated (red) and down-regulated (blue) in INFs vs. NNFs. (**D**) Co-expression network dendrogram with traits for NF-PitNEts. (**E**) Signed association of module eigengenes with the diagnosis. Positive/negative values indicate genes in these modules are up-regulated/down-regulated in INFs compared to NNFs. The log10 (adj. P) values of the six significantly invasiveness-related modules are shown in the right. (**F**) GO term enrichment for genes in blue and turquoise modules. (**G**) Heatmap of correlations between significantly associated module eigengenes.

Our data showed that tumor invasion was correlated with a more aggressive clinical behavior; therefore, we first compared the differential gene expression pattern between NNF- and INF-PitNEts. Both unsupervised hierarchical clustering and sample correlations revealed two main clusters, regardless of the methods employed (i.e. the common method used for clustering and the Pearson method used for correlations). One cluster was significantly enriched for NNFs and the other for INFs, and only a small size (4 INFs and 7 NNFs) of samples were intermingled (Chi-squared test *P* value = 1.8e-05; Figure 2A). Compared to NNF-PitNEts, 843 genes showed increased expression while 1,435 showed decreased expression in INF samples (FDR < 0.05 and │fold change│>2, Figure 2B, Figure S1A and Table S3). These data underscored the presence of characteristic transcriptional profiles in NNF- and INF-PitNEts. Among the 2,278 transcripts, we identified known invasion-related markers, such as CDKN1A (p21) and EZR. Remarkably, differentially expressed genes (DEGs) identified in this study and those from a previous microarray dataset 22 were highly concordant (Figure S1B). Functional annotation of the DEGs revealed significant enrichment for the 'transcription activity', 'ion channel activity', 'plasma membrane' and 'extracellular matrix' networks (Figure 2C, Figure S1C-D).

### Co-expression network analysis to evaluate invasion-associated modules in NF-PitNEts

To find invasiveness-specific expression coregulation, we performed weighted gene correlation network analysis (WGCNA) in the scope of the top 8,000 variant genes and found 12 modules in total, which were identified by color (Figure 2D-E and Figure S2A). Six significantly different modules of genes associated with invasiveness were evident (Figure 2E and Table S4), of which three were upregulated and three downregulated. The most significantly regulated modules, the blue and turquoise modules, were also the largest ones consisting of 1214 and 1540 genes respectively. Given the nature of the data set, both the upregulated and downregulated modules had significant correlations with clinical traits such as 'Gender', 'Texture', and 'Prognosis' (Figure S2B-C). The blue module was enriched in cancer and transcription regulatory genes while the turquoise module was enriched in genes associated with the cell membrane and cell junction (Figure 2F), and the upregulated and downregulated modules exhibited significant negative correlation (Figure 2G).

**Figure 3:**
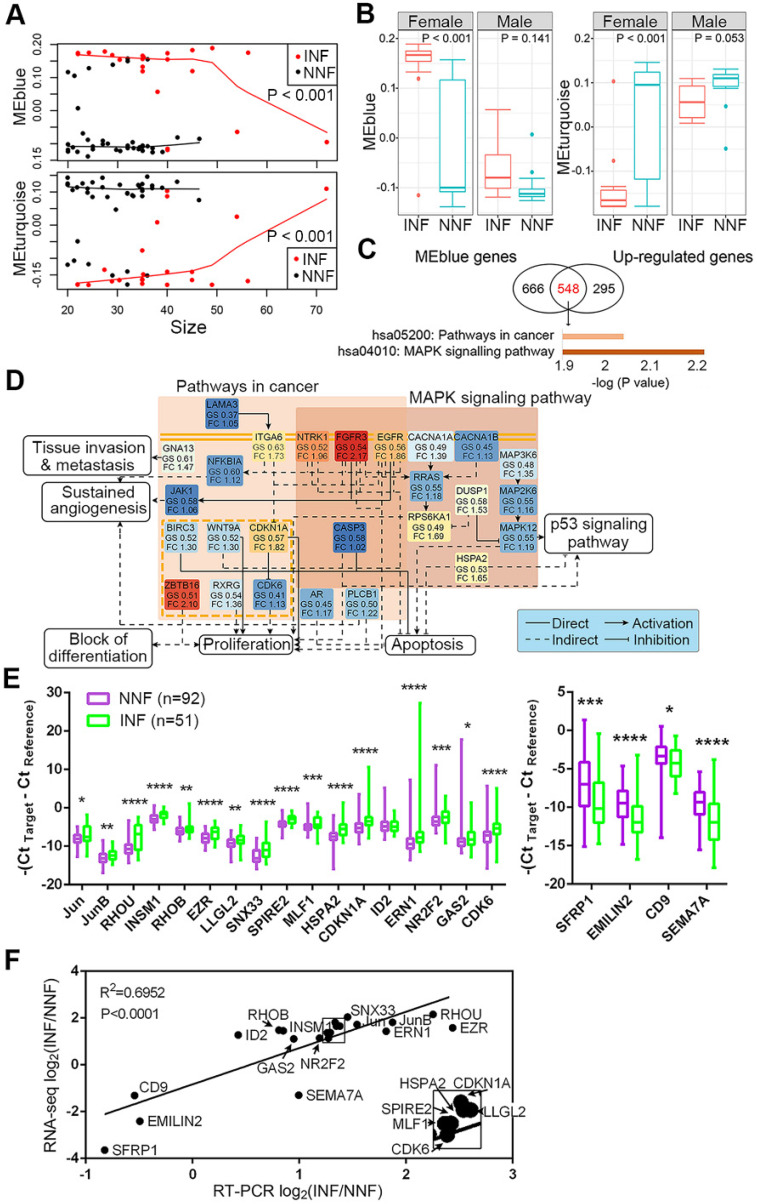
** Candidate DEG identification and their expression between NNF- and INF-PitNEts.** (**A**) Plot of blue and turquoise module eigengenes across tumor size. (**B**) Box plot of module eigengenes of INFs and NNFs between females and males. (**C**) Significantly changed pathways enriched by the overlapped DEGs of blue module and up-regulated genes in INF-PitNEts. (**D**) Representative network of pathways shown in C. GS, gene significances; FC, fold change. The different colors represent their fold-changes. (**E**) Taqman RT-qPCR of up-regulated (left) and down-regulated (right) mRNA expression in INFs (n = 51) compared with NNFs (n = 92). Data are shown for maximum value, 75th percentile, 50th percentile, 25th percentile and minimum value. (**F**) Correlation between the fold-changes of RNA-seq and RT-qPCR analyzed mRNA. Transcripts in the box are magnified in the lower right. *P < 0.05, **P < 0.01, ***P < 0.001,****P < 0.0001, Mann-Whitney U test.

We further analyzed differences in clinical features within each module between NNF- and INF-PitNEts. Although the medium size of INFs was larger than NNFs, the tumor-growth trajectories of NNFs and INFs were different in all significantly regulated modules when tumor sizes matched (Figure 3A and Figure S2D). Additionally, in all modules we identified significant transcriptional changes only among female patients (Figure 3B and Figure S2E), suggesting that gender was closely related to the molecular phenotype.

### Validation of candidate gene expression in NF-PitNEt samples

To further identify functional pathways that discriminated NNFs and INFs, we first intersected 1214 genes in the blue module and 843 upregulated DEGs for INF-PitNEts (Figure 3C). This analysis identified a highly interconnected network of alterations belonging to the pathway in cancers and mitogen-activated protein kinase (MAPK) signaling pathway (Figure 3D). Several receptor tyrosine kinases (eg, EGFR, EGFR3, and NTRK1), their downstream signal transducers (eg, RRAS and JAK1), kinases, and other components showed enhanced expression in INF-PitNEts to jointly regulate several aspects of tumorigenesis. For example, upregulated CDKN1A and CDK6 expression may impair RB-mediated cell cycle control 39. Likewise, 1540 genes in the turquoise module and 1435 downregulated DEGs for INFs were also overlapped and enriched in ECM-receptor interaction and cell adhesion molecules pathways (Figure S3A).

DEGs in various pathways were ranked according to their fold-change between NNFs and INFs and P-value (padj). The top 10-15 genes in each pathway were further manually curated for their reported functions in tumor development. Finally, 17 upregulated and 4 downregulated candidates (Figure S3B) were subjected to qRT-PCR analysis to validate their expression in another cohort of NF-PitNEt samples (Table S5). The differential expression of 20 genes was verified in INFs compared with NNFs (Figure 3E). We also confirmed the consistency of RNA-seq and qRT-PCR results in independent cohorts (Figure 3F).

### *In situ* and circulating INSM1 expression predicts INF-PitNEts

**Figure 4:**
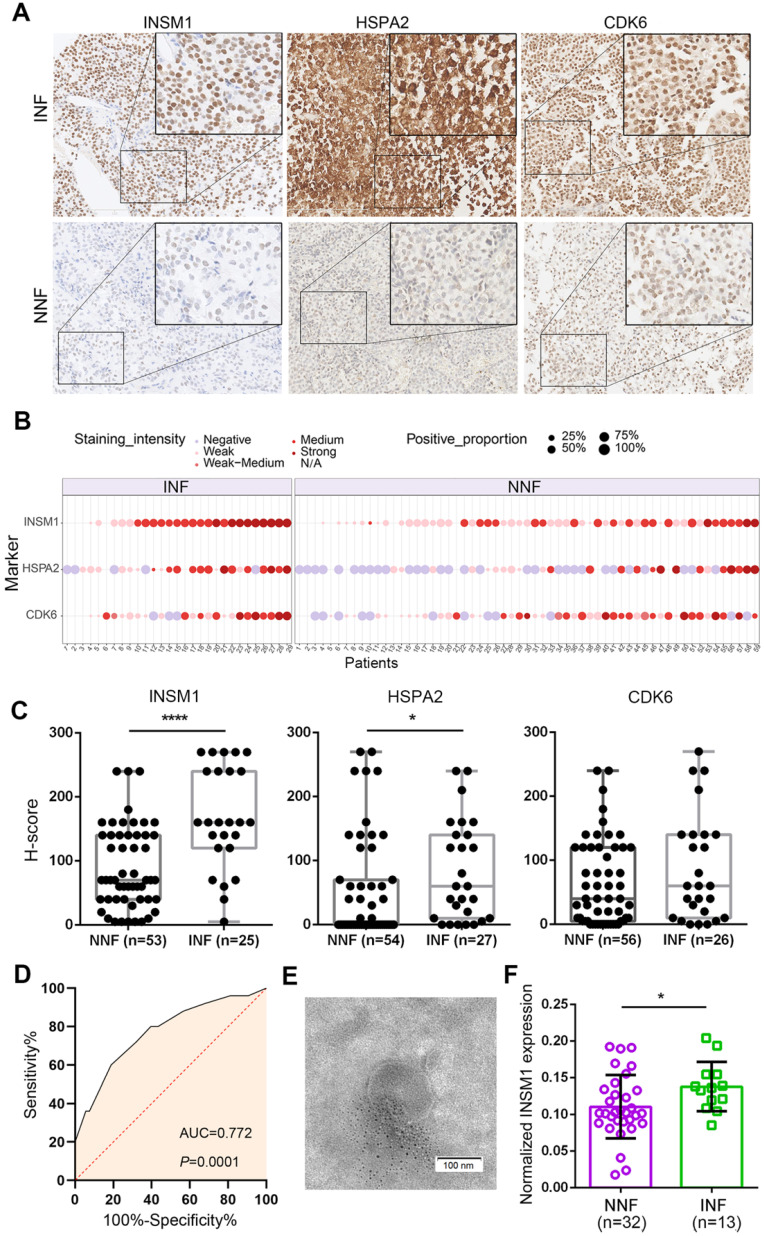
** Detection of *in-situ* and exosomal INSM1 expression and their association with INF-PitNEt diagnostic.** (**A**) Representative images of tissue microarray (TMA) analysis for INSM1, HSPA2, and CDK6 protein expression in NNF and INF samples. INSM1 exhibit specific nuclear immunostaining while HSPA2 and CDK6 display both nuclear and cytoplasmic staining. Original magnification, 200x or 400x; Bar, 200 µm. (**B**) Distribution of INSM1, HSPA2, and CDK6 staining in NF- PitNEt samples. (**C**) The score was obtained by computing staining intensity (0-3) and the proportion of pituitary cells stained for each intensity to give a value between 0 and 300. (**D**) ROC curve showing the true positive and false positive rates for INSM1 immunohistochemical staining. (**E**) Representative transmission images of purified exosomes using the negative staining method. (**F**) Serum-exosomal INSM1 mRNA relative expression in NNF compared with INF samples. *P < 0.05, ****P < 0.0001, Mann-Whitney U test.

To develop INF-PitNEt markers, we first analyzed HSPA2, CDK6, and INSM1 protein expression in a blinded fashion in NNF and INF samples using a tissue microarray (TMA). The three antigens were selected due to their key roles 39-42 in invasion-related dysregulated pathways (Figure 3D), their highly reproducible mRNA differences between RNA-seq and qRT-PCR analyses (Figure 3F), and the reliability of their commercial antibodies. Specific nuclear INSM1 immunoreactivity was observed in NF-PitNEt tissues, whereas HSPA2 and CDK6 were detected in both nuclear and cytoplasmic compartments with CDK6 displaying stronger nuclear staining (Figure 4A). Higher INSM1 and HSPA2 expression was significantly correlated with INFs, with INSM1 showing strong or moderate positive staining in 80% of INF samples but only 42% of NNF samples. Immunopositivity of HSPA2 was demonstrated in 81% of INF samples but only 44% of NNF samples (Figure 4A-C and Figure S4A). In contrast, CDK6 did not show a significant difference between NNFs and INFs (Figure 4A-C and Figure S4A), indicating the possibility of posttranscriptional or translational regulation. The diagnostic performance of INSM1 was shown by receiver operating characteristic (ROC) curves, with the area under the curve (AUC) of 0.772 (95% confidence interval [CI], 0.656-0.887; *P*=0.0001) (Figure 4D). At a cutoff value of 150, sensitivity was 60.0% and specificity was 81.1%.

These results prompted us to further investigate whether serum INSM1 mRNA expression was correlated with NF-PitNEt invasion. We isolated exosomes from serum samples of NF-PitNEt patients using precipitation method, and assessed the morphology and size using transmission electron microscopy and nanoparticle tracking analysis. The average size of exosomes ranged from 140.5 to 183.9 nm with a distinct membrane structure (Figure 4E and Figure S4B). The ddPCR using GAPDH as the normalization control identified significantly higher INSM1 mRNA expression in INFs compared with NNFs (Figure 4F), and the AUC of exosomal INSM1 was 0.719 (95% CI, 0.563-0.874; *P*=0.0227) (Figure S4C). Collectively, these data indicated the predictive value of INSM1 as an *in-situ* or circulating biomarker for INF patients.

### Outliers share transcriptomic similarity with their respective groups

Despite the unique genetic signatures present in INFs versus NNFs, minor overlap still existed (Figure 2A). To analyze the transcriptomic patterns of the 7 NNFs that mingled with INF cluster (NNF outliers) or the 4 INFs that mingled with NNF cluster (INF outliers), *P* value distributions were generated by comparing transcripts observed in outliers with those present either in INFs or NNFs. Remarkably, we observed more genes with lower *P* values than with higher *P* values in the NNF outliers vs. NNF group, which, when viewed together with more differentially expressed genes between these two groups indicated more significant differential expression signals than those between NNF outliers and INFs (Figure S5A-B). Similar results were obtained for INF outliers (Figure S5C-D). Additionally, transcriptional changes between NNF and INF samples showed high concordance between NNF outliers and NNFs (R^2^ = 0.849) or INF outliers and INFs (R2 = 0.844; Figure S5E-F).

Given the high extent of sharing between the outliers and the samples that clustered together, we re-classified NNF outliers as INFs, and INF outliers as NNFs with respect to the transcriptional profiles. As expected, DEGs were highly concordant before or after adjustment, in which 97% of the DEGs before adjustment could be identified after adjustment (Figure S5G-H). These data demonstrated the alternative grouping of NF-PitNEts by transcriptomic profiles, consistent with the notion that marker genes expression and invasion capacity may be independent 10, 11. Nonetheless, these re-classified NNFs had a worse prognosis compared with clinically defined NNFs due to inadequate resection during operation or acquired aggressive traits by radiotherapy (Table 1).

### mRNA profiling combined with tumor subtype allows for INF-PitNEt diagnostics

**Figure 5:**
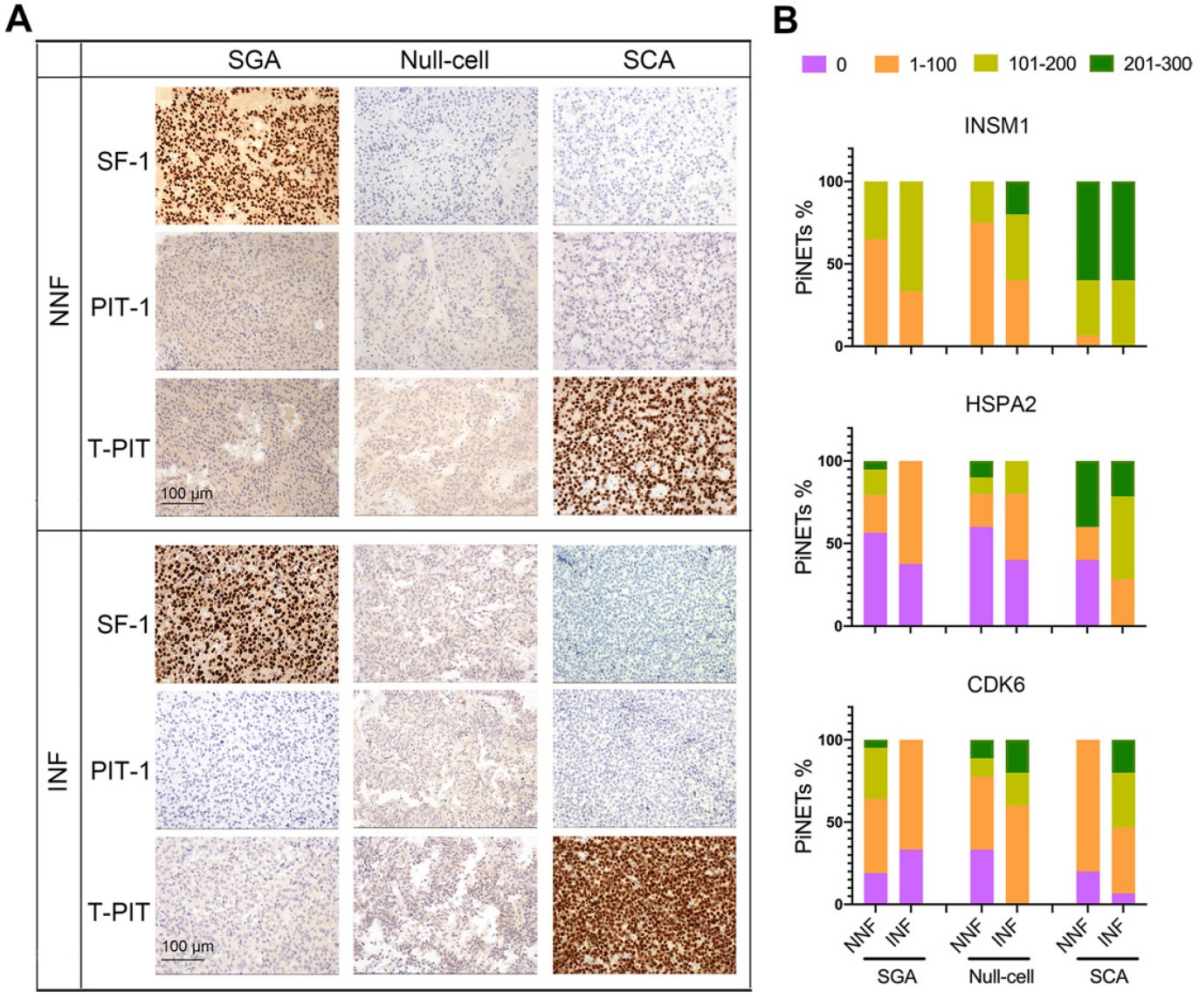
** Expression of INSM1, HSPA2, and CDK6 proteins in NF-PitNEts with different cell lineages.** (**A**) Representative images of immunohistochemistry for transcription factors SF-1, PIT-1, and T-PIT in NNF and INF samples. Original magnification, 200x; Bar, 100 µm. (**B**) Statistical results of INSM1, HSPA2, and CDK6 immunostaining in SGA, null cell adenomas, and SCAs.

Next, we asked whether tumor subtypes contributed to the invasive potential of NF-PitNEts. Based on the latest WHO classification system, NF-PitNEt samples were classified according to adenohypophyseal hormones and transcription factor immunostaining (Figure S5I and Table S1) and validated at the RNA level (Figure S6A). Distinct nuclear staining of SF-1 or T-PIT could be observed independent of the invasive property of NF-PitNEts (Figure 5A). Moreover, an examination of somatostatin receptors (SSTRs) showed lower SSTR2/SSTR3 expression in SCAs (Figure S6A), indicating a higher proliferation and survival potential of these tumors 43, 44. The three subtypes of NF-PitNEts in this study displayed clinical characteristics comparable with previous reports 45, with SCAs showing young age, female gender, and invasion predominance but comparable tumor sizes (Figure S6B-E).

We compared the invasion-related molecular markers in various subtypes of NF-PitNEts and found that HSPA2 and INSM1 expression was higher in SCAs than SGAs or null cell adenomas (Figure 5B, P < 0.0001). For INSM1, the percentage of samples with H-score > 100 was 94.74% (18/19) in SCAs and 38.98% (23/59) in the other two types. The percentage of HSPA2 H-score > 100 was 63.16% (12/19) in SCAs but only 17.74% (11/62) in other types. These results further supported the higher invasive potential of SCAs. We next analyzed the correlation between HSPA2 and INSM1 markers and invasion in each histological type. The moderate to strong INSM1 immunostaining was significantly increased in INFs compared with NNFs only among null cell adenomas (Figure 5B, Table S6), but there was no significant association between HSPA2 protein expression and tumor invasion in each NF-PitNETs subtype (Figure 5B, Table S7). In contrast, CDK6 expression was inversely related to invasiveness in SGAs but showed a tendency of positive correlation with invasive SCA (Figure 5B, Table S8). Similar results were obtained in NF-PitNET subtypes for the 20 marker genes (Figure S6F). Our results indicated that these markers were associated with invasion when all subtypes were considered together.

**Figure 6:**
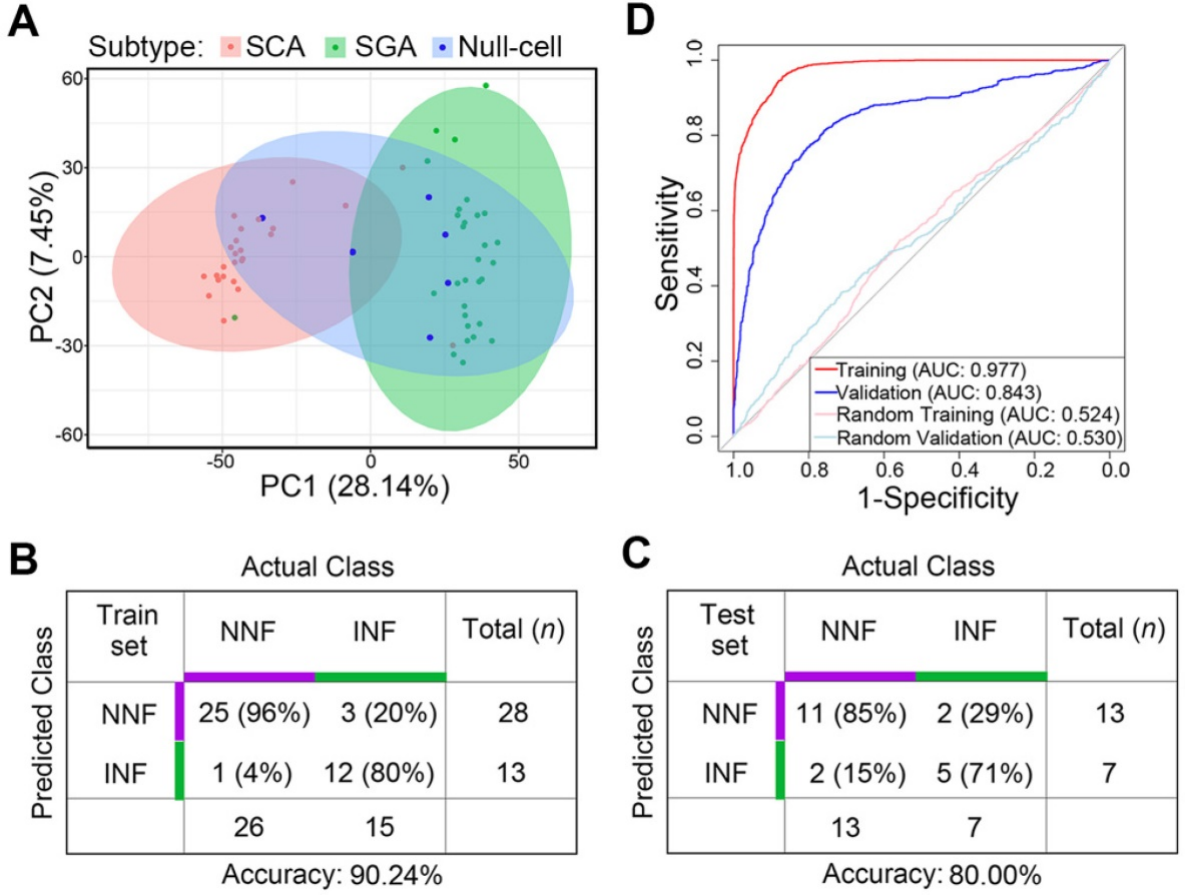
** Differentiating potential of the gene-subtype panel for INF- and NNF-PitNEts.** (**A**) PCA with the top 5000 variant genes of the 61 NF-PitNEt samples. (**B and C**) Performance of the 20-gene-subtype SVM algorithm in the training cohort (B) and test cohort (C). Sample numbers and detection rates in percentages are shown. (**D**) ROC-curve of SVM diagnostics of training (red) and validation (blue) cohorts indicating the classification accuracies obtained by chance of the training and validation cohort (gray) in RNA-seq data.

Principal-component analysis (PCA) analysis revealed that SCAs and SGAs were clustered into distinct groups while null cell adenomas exhibited both SCA and SGA transcriptomic features (Figure 6A). Also, previously identified NNF outliers were all SCAs and 2 out of the 3 INF outliers were SGAs, suggesting that cell lineage was a covariate for tumor invasiveness.

Finally, we tested the predictive potential of the 20 validated DEGs between NNFs and INFs in combination with tumor subtypes, utilizing a support vector machine (SVM) algorithm with leave-one-out cross validation (LOOCV). In brief, we trained the classifier from N - 1 where N denotes the number of subjects and we predicted the invasiveness of the Nth. The entire approach was repeated for N times to predict the invasiveness of each sample in the cohort. Among the RNA-seq group, the accuracy was 90.24% for the N = 41 training cohort (Figure 6B). Subsequent validation in the other 20 samples yielded an accuracy of 80% (Figure 6C), with an AUC of 0.843 to differentiate INFs from NNFs (Figure 6D). These results showed that, although the sample size was relatively small, the 20-gene-subtype classifier displayed significant strength to diagnose invasive NF-PitNEts.

## Discussion

Adenoma invasion and cell proliferation (mitotic count and Ki-67 index) are two important prognostic features for tumor recurrence. Since the correlation between the proliferation marker, Ki-67 and tumor invasion was not always consistent 46, the two features should be judged separately. The diagnosis of NF-PitNEts is based solely on the morphologic features as their hormonal activity is clinically undetectable 15; hence, accurate detection of these invasive tumors at early stages remains the focus of much research. Both RNA and protein analyses have identified activated Wnt and Notch pathways but suppressed TGF-β/Smad signaling in the progression of NF-PitNEts 47, 48. Also, up-regulation of several markers in INF-PitNEts, including myosin 5A (MYO5A) 23, EZR 22, hsa-miR-181a-5p 21 and angiogenic factor VEGF 49 has been reported. These studies, despite relatively small sample size, showed the promise of developing biomarkers for INF-PitNEts.

Our RNA-seq data displayed distinct mRNA features between NNF- and INF-PitNEts, and more than 95% of the qRT-PCR-validated genes were consistent with sequencing results. Among them, INSM1 and HSPA2 exhibited strong concordance in their mRNA and protein expression. HSPA2 (Hsp70-2), a homolog of Hsp70, is essential for the physiology of spermatogenesis and is also involved in the pathology of several types of tumors by promoting cell cycle progression and angiogenesis 42, 50; however, its function in PitNEt is still unclear. The second marker INSM1, a zinc finger transcription factor is implicated in the differentiation of endocrine cells in the pituitary and other tissues 51, and its expression is significantly increased in multiple neuroendocrine neoplasms. Our finding of 100% positive INSM1 immunostaining in NF-PitNEts is in line with the results from the central nervous system neoplasms 52 and neuroendocrine tumors 53. Recent studies have demonstrated that INSM1 staining outperforms conventional markers for identifying and grading neuroendocrine tumors 40, 54, 55. Our results revealed that INSM1 is also a highly sensitive diagnostic marker for tumor invasion in non-functioning pituitary neuroendocrine tumors, with strong nuclear staining and homogenous expression pattern. It is worth investigating whether INSM1 could similarly distinguish between invasive and non-invasive functioning PitNEts.

So far, surgery and/or radiotherapy are the first-line treatment options for NF-PitNEts, and the biomarkers allowing early detection of NF-PitNEts invasiveness are scarce. The potential of circulating RNAs as biomarkers in blood has been implicated in the diagnosis, prognosis, and recurrence of PitNEts 56, 57. In the current study, serum-exosomal INSM1 mRNA demonstrated moderate sensitivity and specificity with an AUC value of 0.719 in distinguishing INFs from NNFs. Thus, we reasoned that the detection of *in-situ* or circulating INSM1 might be an effective and better substitute for the existing invasive detection strategies.

The current findings of the clinical characteristics of NF-PitNEt subtypes are largely controversial 45, because they relied on inconsistent histopathological indices to classify NF-PitNEts. Here, we defined NF-PitNEts according to the criteria of the most recent WHO system, and confirmed that 66% of both ACTH-positive and ACTH-negative SCAs, but only 10% of SGAs and 33% of null cell adenomas, had invasive potential, and SCAs presented at an earlier age than other subtypes (47.6 vs. 54.3 yrs old). Also, higher SSTR2/SSTR3 expression in SCAs indicated the potential efficacy of somatostatin analogs for these tumors 58.

The transcriptomic heterogeneity among NNF or INF patients could be justified by their gonadotroph or corticotroph cells of origin. SCAs and SGAs displayed relatively distinct transcriptomic characteristics. Notably, although null cell adenomas and SGAs were previously recognized by different behavioral characteristics 13, these two subtypes shared transcriptomic similarity. A recent comprehensive multi-omics study by Neou *et al.* 19 also obtained similar results, with gonadotroph and null cell tumors clustering together based on the transcriptome. Our results also showed that invasion-related markers were unable to differentiate invasive tumors within SCAs and SGAs. These findings suggested that the invasion-associated marker gene expression is connected to a potential subtype(s) of PitNEts.

In summary, the NF-PitNEt classification yielded a gene-subtype panel that predicted INF with high accuracy. Since IHC staining for transcription factors was only available among RNA-seq and TMA samples in this study, the potential of this panel for differentiating invasiveness needs to be confirmed in a larger cohort of patients. Nevertheless, we have shown that the precise classification of NF-PitNEts, in combination with the RNA profile, could potentially aid in predicting the disease course.

## Supplementary Material

Supplementary figures and tables.

Supplementary table S3.

## Figures and Tables

**Table 1:** Clinical characteristics, surgical results, postoperative complications, and prognosis of NNF- and INF-PitNEt patients for RNA-seq

Variable	NNF	INF	*p* value
**Gender**			
Female (%)	21 (53.8)	18 (81.8)	**0.029**
Male (%)	18 (46.2)	4 (18.2)	
Age (years)	55.2±10.6	47.2±10.2	**0.006**
Height (cm)	164.0±6.6	162.3±8.2	0.380
Weight (kg)	66.6±10.8	67.5±13.8	0.779
Maximum diameter of tumor (mm)	29.2±6.5	39.7±12.0	**0.0007**
**Degree of invasion**			
Knosp Grade 0 (%)	16 (41.0)	0 (0)	**<0.001**
Knosp Grade 1-2 (%)	23 (59.0)	0 (0)	**<0.001**
Knosp Grade 3 (%)	0 (0)	0 (0)	
Knosp Grade 4 (%)	0 (0)	22 (100.0)	**<0.001**
**Extent of tumor resection**			
GTR (%)	29 (74.4)	0 (0)	**<0.001**
STR (%)	10 (25.6)	10 (45.5)	0.270
PR (%)	0 (0)	12 (54.5)	**<0.001**
**Ki-67**	1.7±0.7	1.7±1.0	1
≤3% (%)	39 (100.0)	21 (95.5)	0.120
>3% (%)	0 (0)	1 (4.5)	
**P53**			
Positive (%)	1 (2.6)	2 (9.1)	0.262
Negative (%)	38 (97.4)	20 (90.9)	
Mean follow-up in months (range)	58.2 (45-72)	60.4 (46-75)	0.162
**Postoperative symptoms**			
Headache (%)	2 (5.1)	7 (31.8)	**0.005**
Visual dysfunction (%)	1 (2.6)	5 (22.7)	**0.011**
Monocular blindness (%)	0 (0)	2 (9.1)	**0.027**
Panhypopituitarism (%)	1 (2.6)	5 (22.7)	**0.011**
Permanent diabetes insipidus (%)	1 (2.6)	1 (4.5)	0.683
Cranial nerve palsy (%)	0 (0)	6 (27.3)	**<0.001**
**Follow-up treatments**			
Radiation therapy (%)	4 (10.3)	20 (90.9)	**<0.001**
Medical therapy (%)	0 (0)	6 (27.3)	**<0.001**
Repeated surgery (%)	3 (7.7)	8 (36.4)	**0.002**
**Tumor control**			
Without tumor (%)	29 (74.4)	0 (0)	**<0.001**
Tumor stability (%)	10 (25.6)	14 (63.6)	**0.004**
Growth of tumor (%)	0 (0)	8 (36.4)	**<0.001**
**Death (%)**	0 (0)	2 (9.1)	**0.027**

GTR = grosstotal resection; PR = partial resection; STR = subtotal resection. Boldface type indicates statistical significance.

## References

[1] Chahal HS, Stals K, Unterländer M, Balding DJ, Thomas MG, Kumar AV (2011). AIP mutation in pituitary adenomas in the 18th century and today. N Engl J Med.

[2] Newey PJ, Nesbit MA, Rimmer AJ, Head RA, Gorvin CM, Attar M (2013). Whole-exome sequencing studies of nonfunctioning pituitary adenomas. J Clin Endocrinol Metab.

[3] Melmed S (2011). Pathogenesis of pituitary tumors. Nat Rev Endocrinol.

[4] Lopes MBS (2017). The 2017 World Health Organization classification of tumors of the pituitary gland: a summary. Acta Neuropathol.

[5] Ntali G, Capatina C, Grossman A, Karavitaki N (2014). Clinical review: Functioning gonadotroph adenomas. J Clin Endocrinol Metab.

[6] Nishioka H, Inoshita N, Mete O, Asa SL, Hayashi K, Takeshita A (2015). The Complementary Role of Transcription Factors in the Accurate Diagnosis of Clinically Nonfunctioning Pituitary Adenomas. Endocr Pathol.

[7] Tischler AS, Pacak K, Eisenhofer G (2014). The adrenal medulla and extra-adrenal paraganglia: then and now. Endocr Pathol.

[8] Knosp E, Steiner E, Kitz K, Matula C (1993). Pituitary adenomas with invasion of the cavernous sinus space: a magnetic resonance imaging classification compared with surgical findings. Neurosurgery.

[9] Di Ieva A, Rotondo F, Syro LV, Cusimano MD, Kovacs K (2014). Aggressive pituitary adenomas-diagnosis and emerging treatments. Nat Rev Endocrinol.

[10] Yokoyama S, Hirano H, Moroki K, Goto M, Imamura S, Kuratsu JI (2001). Are nonfunctioning pituitary adenomas extending into the cavernous sinus aggressive and/or invasive?. Neurosurgery.

[11] Scheithauer BW, Kovacs KT, Laws ER, Randall RV (1986). Pathology of invasive pituitary tumors with special reference to functional classification. J Neurosurg.

[12] Inoshita N, Nishioka H (2018). The 2017 WHO classification of pituitary adenoma: overview and comments. Brain Tumor Pathol.

[13] Balogun JA, Monsalves E, Juraschka K, Parvez K, Kucharczyk W, Mete O (2015). Null cell adenomas of the pituitary gland: an institutional review of their clinical imaging and behavioral characteristics. Endocr Pathol.

[14] Raverot G, Burman P, McCormack A, Heaney A, Petersenn S, Popovic V (2018). European Society of Endocrinology Clinical Practice Guidelines for the management of aggressive pituitary tumours and carcinomas. Eur J Endocrinol.

[15] Asa SL, Ezzat S (2009). The pathogenesis of pituitary tumors. Annu Rev Pathol.

[16] Knosp E, Kitz K, Perneczky A (1989). Proliferation activity in pituitary adenomas: measurement by monoclonal antibody Ki-67. Neurosurgery.

[17] Gupta P, Dutta P (2018). Landscape of Molecular Events in Pituitary Apoplexy. Front Endocrinol (Lausanne).

[18] Vallar L, Spada A, Giannattasio G (1987). Altered Gs and adenylate cyclase activity in human GH-secreting pituitary adenomas. Nature.

[19] Neou M, Villa C, Armignacco R, Jouinot A, Raffin-Sanson ML, Septier A (2020). Pangenomic Classification of Pituitary Neuroendocrine Tumors. Cancer Cell.

[20] Aydin B, Arga KY (2019). Co-expression Network Analysis Elucidated a Core Module in Association With Prognosis of Non-functioning Non-invasive Human Pituitary Adenoma. Front Endocrinol (Lausanne).

[21] Wu S, Gu Y, Huang Y, Wong TC, Ding H, Liu T (2017). Novel Biomarkers for Non-functioning Invasive Pituitary Adenomas were Identified by Using Analysis of microRNAs Expression Profile. Biochem Genet.

[22] Chen Y, Chuan H-L, Yu S-Y, Li C-Z, Wu Z-B, Li G-L (2017). A Novel Invasive-Related Biomarker in Three Subtypes of Nonfunctioning Pituitary Adenomas. World Neurosurg.

[23] Galland F, Lacroix L, Saulnier P, Dessen P, Meduri G, Bernier M (2010). Differential gene expression profiles of invasive and non-invasive non-functioning pituitary adenomas based on microarray analysis. Endocr Relat Cancer.

[24] Micko AS, Wohrer A, Wolfsberger S, Knosp E (2015). Invasion of the cavernous sinus space in pituitary adenomas: endoscopic verification and its correlation with an MRI-based classification. J Neurosurg.

[25] Harrow J, Frankish A, Gonzalez JM, Tapanari E, Diekhans M, Kokocinski F (2012). GENCODE: the reference human genome annotation for The ENCODE Project. Genome Res.

[26] Trapnell C, Roberts A, Goff L, Pertea G, Kim D, Kelley DR (2012). Differential gene and transcript expression analysis of RNA-seq experiments with TopHat and Cufflinks. Nat Protoc.

[27] Anders S, Pyl PT, Huber W (2015). HTSeq-a Python framework to work with high-throughput sequencing data. Bioinformatics.

[28] Sugimachi K, Matsumura T, Hirata H, Uchi R, Ueda M, Ueo H (2015). Identification of a bona fide microRNA biomarker in serum exosomes that predicts hepatocellular carcinoma recurrence after liver transplantation. Br J Cancer.

[29] Love MI, Huber W, Anders S (2014). Moderated estimation of fold change and dispersion for RNA-seq data with DESeq2. Genome Biol.

[30] Langfelder P, Horvath S (2008). WGCNA: an R package for weighted correlation network analysis. BMC Bioinformatics.

[31] Huang DW, Sherman BT, Lempicki RA (2009). Systematic and integrative analysis of large gene lists using DAVID bioinformatics resources. Nat Protoc.

[32] Subramanian A, Tamayo P, Mootha VK, Mukherjee S, Ebert BL, Gillette MA (2005). Gene set enrichment analysis: a knowledge-based approach for interpreting genome-wide expression profiles. Proc Natl Acad Sci USA.

[33] Best MG, Sol N, Kooi I, Tannous J, Westerman BA, Rustenburg F (2015). RNA-Seq of Tumor-Educated Platelets Enables Blood-Based Pan-Cancer, Multiclass, and Molecular Pathway Cancer Diagnostics. Cancer Cell.

[34] Robin X, Turck N, Hainard A, Tiberti N, Lisacek F, Sanchez J-C (2011). pROC: an open-source package for R and S+ to analyze and compare ROC curves. BMC Bioinformatics.

[35] Bujko M, Rusetska N, Mikula M (2016). Validating candidate reference genes for qRT-PCR-based gene expression analysis in nonfunctioning pituitary adenomas. Pituitary.

[36] Vandesompele J, De Preter K, Pattyn F, Poppe B, Van Roy N, De Paepe A (2002). Accurate normalization of real-time quantitative RT-PCR data by geometric averaging of multiple internal control genes. Genome Biol.

[37] Wallace TJ, Qian J, Avital I, Bay C, Man Y-G, Wellman LL (2018). Technical Feasibility of Tissue Microarray (TMA) Analysis of Tumor-Associated Immune Response in Prostate Cancer. J Cancer.

[38] Hirsch FR, Varella-Garcia M, Bunn PA, Di Maria MV, Veve R, Bremmes RM (2003). Epidermal growth factor receptor in non-small-cell lung carcinomas: correlation between gene copy number and protein expression and impact on prognosis. J Clin Oncol.

[39] Paternot S, Bockstaele L, Bisteau X, Kooken H, Coulonval K, Roger PP (2010). Rb inactivation in cell cycle and cancer: the puzzle of highly regulated activating phosphorylation of CDK4 versus constitutively active CDK-activating kinase. Cell Cycle.

[40] Chen C, Notkins AL, Lan MS (2019). Insulinoma-Associated-1: From Neuroendocrine Tumor Marker to Cancer Therapeutics. Mol Cancer Res.

[41] Zhang T, Liu W-D, Saunee NA, Breslin MB, Lan MS (2009). Zinc finger transcription factor INSM1 interrupts cyclin D1 and CDK4 binding and induces cell cycle arrest. J Biol Chem.

[42] Daugaard M, Jäättelä M, Rohde M (2005). Hsp70-2 is required for tumor cell growth and survival. Cell Cycle.

[43] Vázquez-Borrego MC, Gupta V, Ibáñez-Costa A, Gahete MD, Venegas-Moreno E, Toledano-Delgado Á (2020). A Somatostatin Receptor Subtype-3 (SST) Peptide Agonist Shows Antitumor Effects in Experimental Models of Nonfunctioning Pituitary Tumors. Clin Cancer Res.

[44] Theodoropoulou M, Stalla GK (2013). Somatostatin receptors: from signaling to clinical practice. Front Neuroendocrinol.

[45] Drummond J, Roncaroli F, Grossman AB, Korbonits M (2019). Clinical and Pathological Aspects of Silent Pituitary Adenomas. The Journal of clinical endocrinology and metabolism.

[46] Amar AP, Hinton DR, Krieger MD, Weiss MH (1999). Invasive pituitary adenomas: significance of proliferation parameters. Pituitary.

[47] Moreno CS, Evans C-O, Zhan X, Okor M, Desiderio DM, Oyesiku NM (2005). Novel molecular signaling and classification of human clinically nonfunctional pituitary adenomas identified by gene expression profiling and proteomic analyses. Cancer Res.

[48] Zhenye L, Chuzhong L, Youtu W, Xiaolei L, Lei C, Lichuan H (2014). The expression of TGF-β1, Smad3, phospho-Smad3 and Smad7 is correlated with the development and invasion of nonfunctioning pituitary adenomas. J Transl Med.

[49] Sánchez-Ortiga R, Sánchez-Tejada L, Moreno-Perez O, Riesgo P, Niveiro M, Picó Alfonso AM (2013). Over-expression of vascular endothelial growth factor in pituitary adenomas is associated with extrasellar growth and recurrence. Pituitary.

[50] Yang Y-L, Zhang Y, Li D-D, Zhang F-L, Liu H-Y, Liao X-H (2020). RNF144A functions as a tumor suppressor in breast cancer through ubiquitin ligase activity-dependent regulation of stability and oncogenic functions of HSPA2. Cell Death Differ.

[51] Welcker JE, Hernandez-Miranda LR, Paul FE, Jia S, Ivanov A, Selbach M (2013). Insm1 controls development of pituitary endocrine cells and requires a SNAG domain for function and for recruitment of histone-modifying factors. Development.

[52] Ames HM, Rooper LM, Laterra JJ, Eberhart CG, Rodriguez FJ (2018). INSM1 Expression Is Frequent in Primary Central Nervous System Neoplasms but Not in the Adult Brain Parenchyma. J Neuropathol Exp Neurol.

[53] Staaf J, Tran L, Söderlund L, Nodin B, Jirström K, Vidarsdottir H (2020). Diagnostic Value of Insulinoma-Associated Protein 1 (INSM1) and Comparison With Established Neuroendocrine Markers in Pulmonary Cancers: A Comprehensive Study and Review of the Literature. Arch Pathol Lab Med.

[54] Mukhopadhyay S, Dermawan JK, Lanigan CP, Farver CF (2019). Insulinoma-associated protein 1 (INSM1) is a sensitive and highly specific marker of neuroendocrine differentiation in primary lung neoplasms: an immunohistochemical study of 345 cases, including 292 whole-tissue sections. Mod Pathol.

[55] Mahalakshmi B, Baskaran R, Shanmugavadivu M, Nguyen NT, Velmurugan BK (2020). Insulinoma-associated protein 1 (INSM1): a potential biomarker and therapeutic target for neuroendocrine tumors. Cell Oncol (Dordr).

[56] Zhang Y, Liu YT, Tang H, Xie WQ, Yao H, Gu WT (2019). Exosome-Transmitted lncRNA H19 Inhibits the Growth of Pituitary Adenoma. J Clin Endocrinol Metab.

[57] Di Ieva A, Butz H, Niamah M, Rotondo F, De Rosa S, Sav A (2014). MicroRNAs as biomarkers in pituitary tumors. Neurosurgery.

[58] Wang QL, Zhuang X, Sriwastva MK, Mu J, Teng Y, Deng Z (2018). The theranostic promise for Neuroendocrine Tumors in the late 2010s - Where do we stand, where do we go?. Theranostics.

