# Supplementary material for: Transcriptomic analysis identifies a tumor subtype mRNA classifier for invasive non-functioning pituitary neuroendocrine tumor diagnostics
Source: Theranostics. 2021 Jan 1;11(1):132–46. doi: 10.7150/thno.47525 (PMC7681103; doi:10.7150/thno.47525)
Supplement: Supplementary file 1 — Supplementary figures and tables. [file thnov11p0132s1.pdf]

Supplementary Figures

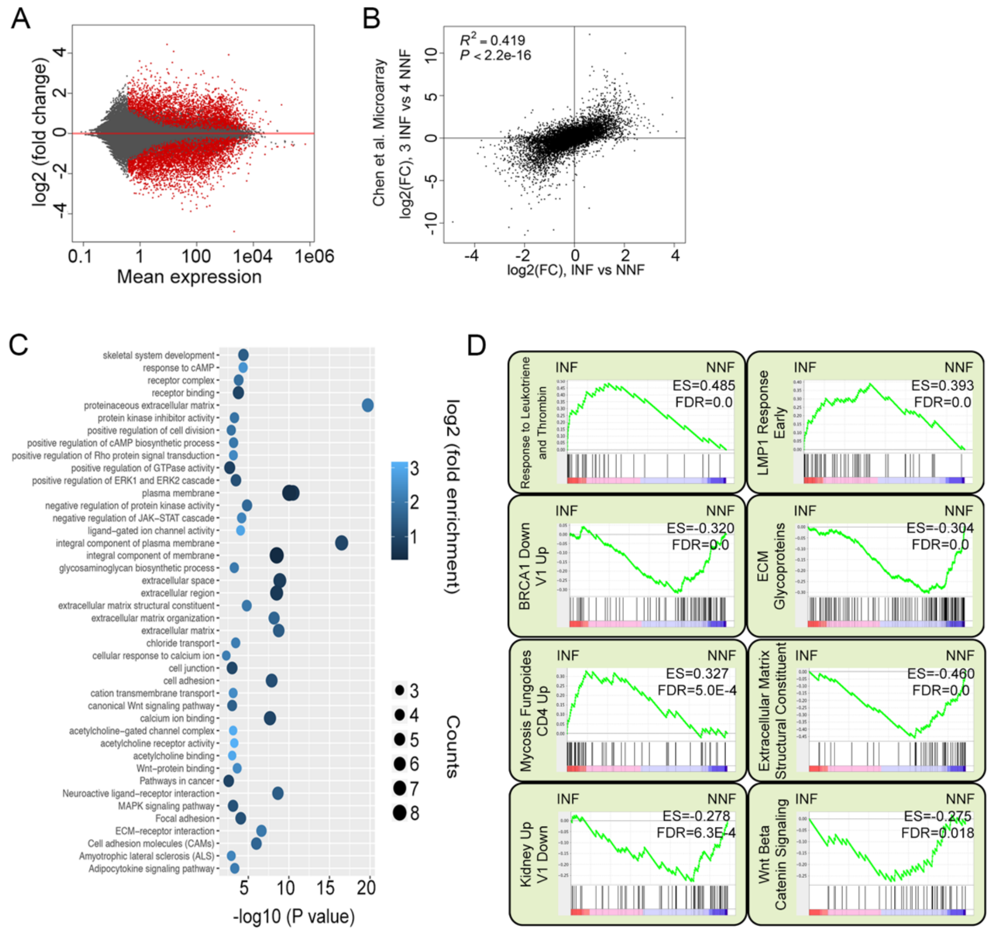

Figure S1. Differential gene expression and pathway analysis in NNF- vs. INF-PitNEts.

(A) MA plot of all genes identified by RNA-seq between NNF- vs. INF-PitNEts. (B) Independent replication analysis of NNF- vs. INF-PitNEt gene fold changes between previously reports from ref. 22 by microarray and data from this study by RNA-seq. The RNA-seq data show a replication signal between previous study and results from this study. (C) GO analysis of differential expression genes in NNF- vs. INF-PitNEt. (D) Gene set enrichment analysis (GSEA) showing characteristic data set from the NNF vs. INF group.

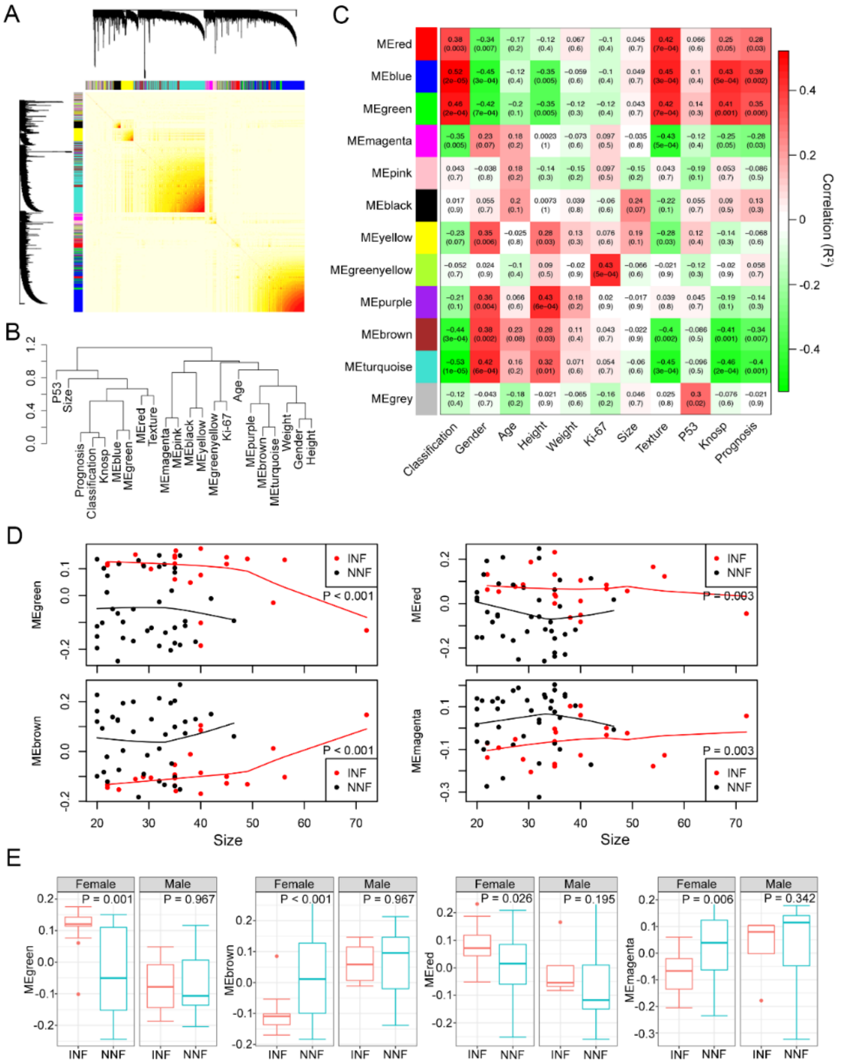

Figure S2. Weighted gene co-expression network analysis of NF-PitNETs.

(A) Heatmap of topological overlap matrix combined with the corresponding hierarchical clustering dendrogram and module colors. (B) Clustering dendrogram of the relationships among the module eigengenes and the clinical traits. (C) Correlation analysis based on the expression of the transcriptomic signature in two independent expression datasets with NNFs and INFs. (D) Plot of green, brown, red and magenta module eigengenes across tumor size. (E) Eigengene expression between sex in green, brown, red and magenta modules. *P* values are for the difference between temporal trajectories for NNFs and INFs by permutation test.

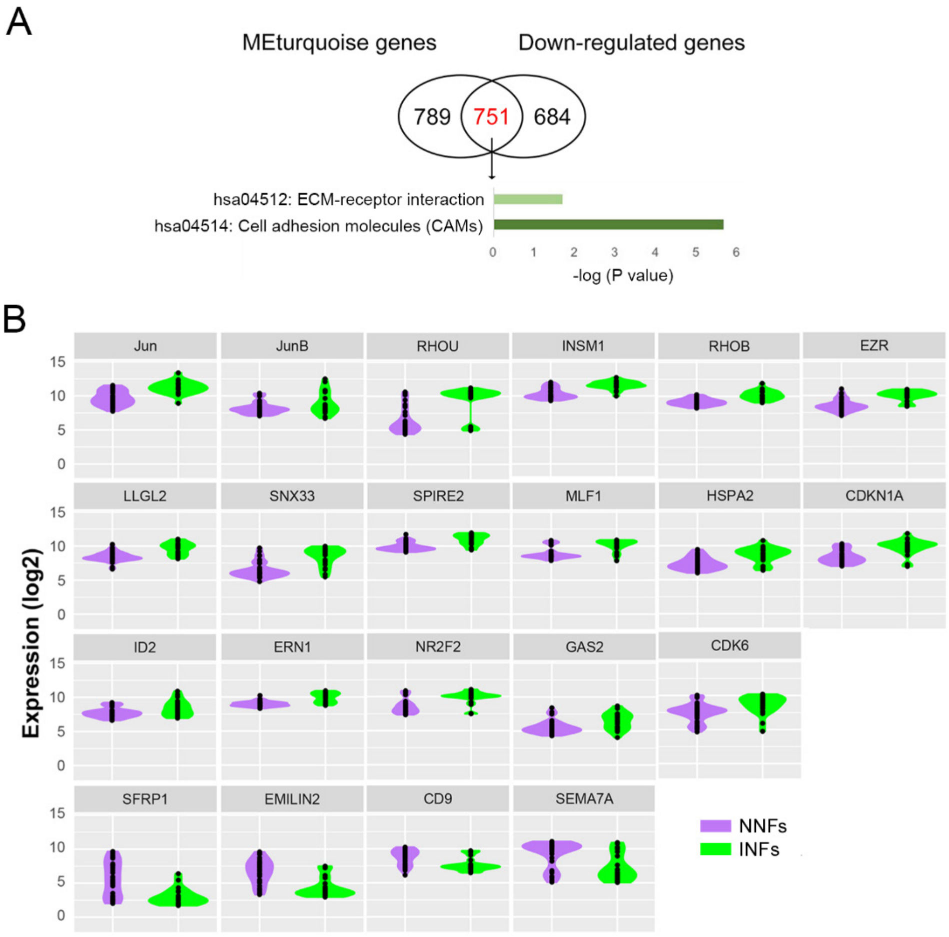

Figure S3. Significantly modulated pathways and genes between NNF- vs. INF-PitNEts.

(A) Significantly changed pathways enriched by the overlapped DEGs of turquoise module and down-regulated genes in INFs. (B) Violin plots representing the expression of DEGs that selected for qPCR validation in NNF vs. INF group.

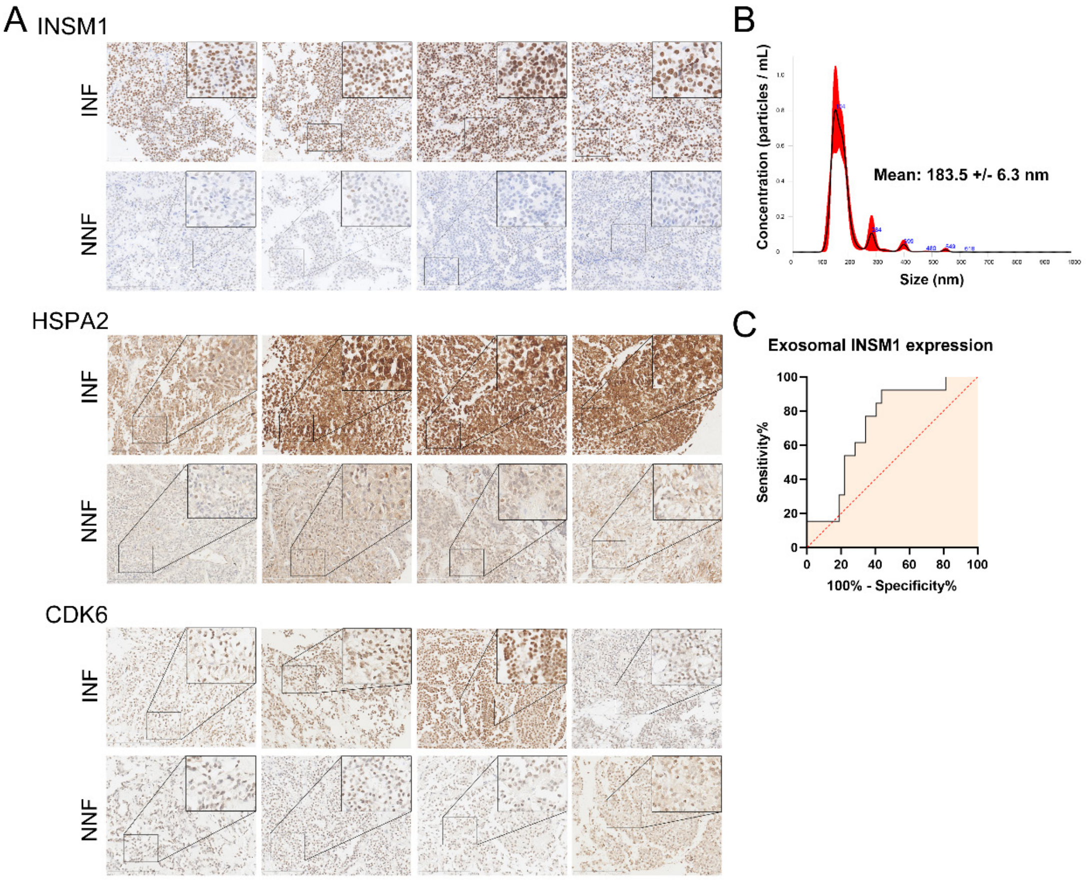

Figure S4. The histological and serum-exosomal INSM1 expression in NF-PitNEts.

(A) Additional figures for TMA analyzing INSM1, HSPA2 and CDK6 expression in NF-PitNEts. Original magnification, 200 x or 400 x; Bar, 200 μm. (B) Size distribution and concentration of exosomes analyzed by the NanoSight NS300 instrument. (C) ROC curve for exosomal INSM1 mRNA. At a cutoff value of 0.104, sensitivity was 56.3% and specificity was 92.3%. AUC, area under the curve.

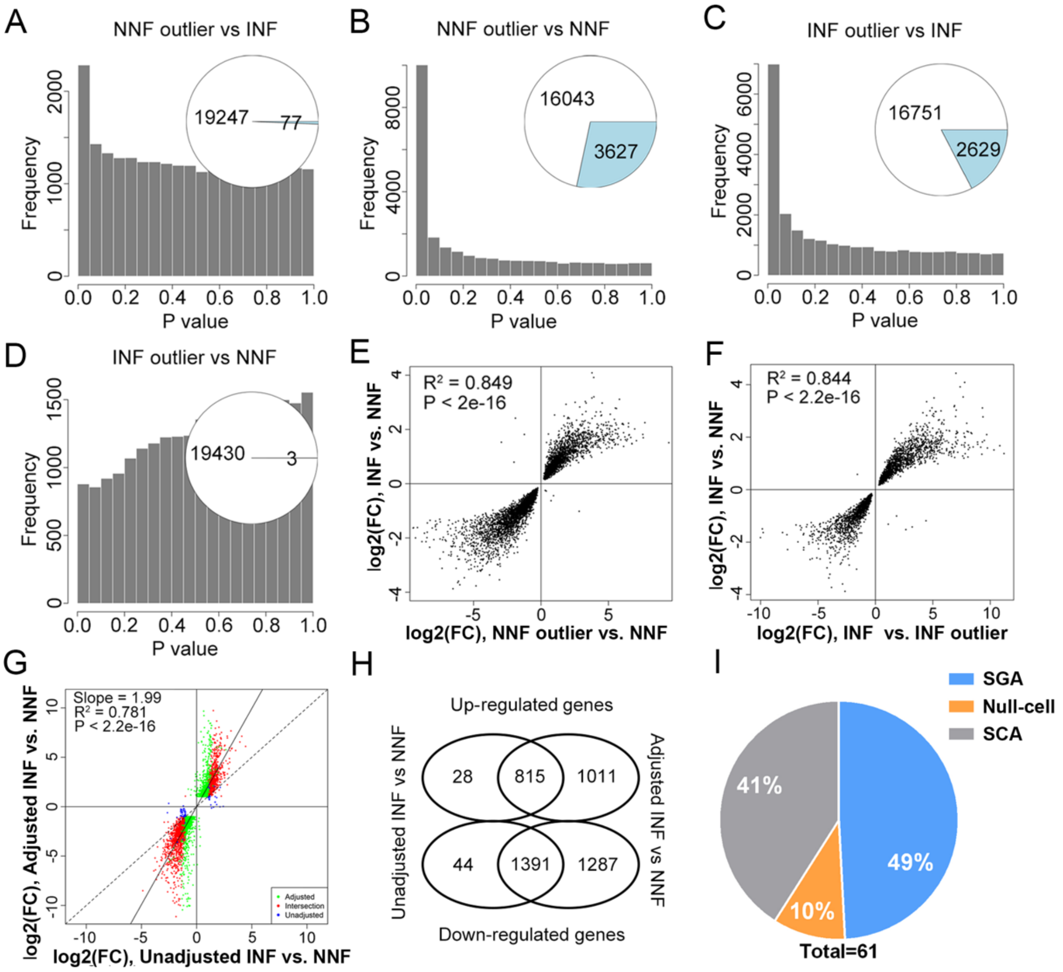

Figure S5. NF-PitNEts were divided into three subtypes.

(A-D) The distribution of *P* values and numbers of gene expression in NNF outliers vs. INFs, NNF outliers vs. NNFs, INF outliers vs. INFs, INF outliers vs. NNFs. (E) Replication of DGEs between NNF outliers vs. NNF and INF vs. NNF. (F) Replication of DGEs between INF outliers vs. INF and INF vs. NNF. (G) Replication of DEGs between outlier-unadjusted INF vs. NNF and outlier-adjusted INF vs. NNF. Red, DEGs in both; green, DEGs only in outlier-adjusted INF vs. NNF; blue, DEGs only in outlier-unadjusted INF vs. NNF. (H) Venn diagram of DEGs in outlier-unadjusted INF vs. NNF and outlier-adjusted INF vs. NNF was presented below. (I) NF-PA subtype distribution.

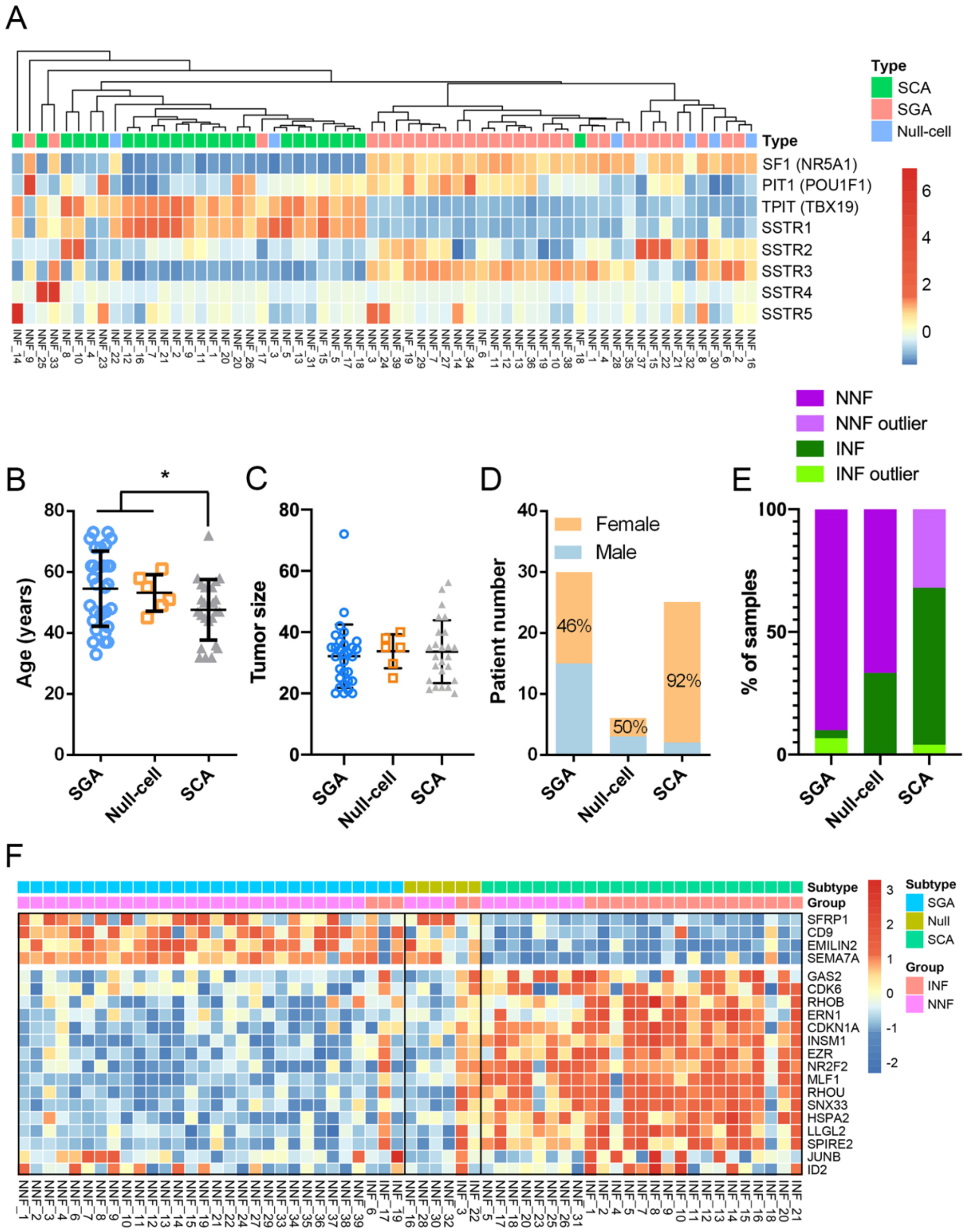

Figure S6. The clinical characteristics of NF-PAs with different cell lineages.

(A) Expression of transcription factors and somatostatin receptors (SSTRs) genes in NF-PA samples from RNA-seq data. (B-E) Identification of the age (B), tumor size (C), gender (D) and invasiveness (E) distribution among gonadotroph, null cell and

corticotroph NF-PAs. (F) Expression of 20 invasion-related markers in SGA, null cell adenoma and SCA. **P*<0.05, ***P*<0.01, ****P*<0.001, ****P*<0.0001, Mann-Whitney U test.

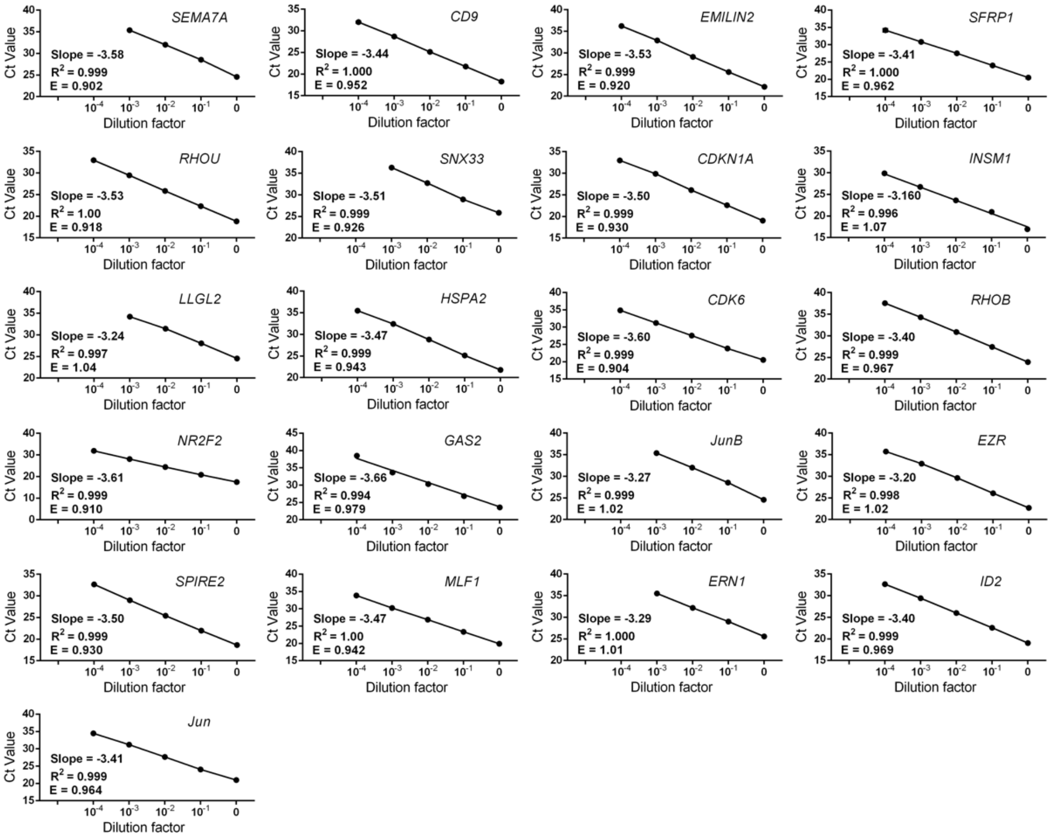

Figure S7. Standard curves for all the validated genes.

Quantification of the pituitary cDNA was performed using specific primer pairs for each gene. Calibration curves generated using 4- or 5-fold dilutions of cDNA samples (triplicates). The Cq values are plotted against the concentration of the nucleic acid target as copies/reaction. To determine the reaction efficiency (E), the slope of each calibration curve was determined from the following equation: $E = 10^{-1}/\text{slope} - 1$.

Supplementary Tables

Table S1. Classification of NF-PitNEts for RNA-seq according to pituitary hormones and transcription factors.

Cell lineage	Pituitary Hormones by IHC	Transcription Factors by IHC	No. NNF	No. INF	Total
Corticotroph Adenomas	ACTH	TPIT	1	2	3
	None	TPIT	7	15	22
Gonadotroph Adenomas	FSHβ and/or LHβ	SF1	7	0	7
	FSHβ and/or LHβ	None	20	3	23
Null cell adenomas	None	None	4	2	6
Total			39	22	61

Table S2. Primers and probes used in qRT-PCR.

Gene name	Gene ID	Forward primer (5'-3')	Reverse primer (5'-3')	Probe (5'-3')
SFRP1	6422	GAGGAGGTTTGCC TGTGATTT	TCCCAGGACTCTA TCCAGAATG	AATTATCGCTAGGGCC AAGGTGGG
EMILIN2	8403 4	CAGCAGCAGTGTG ATGACTAT	CGGGCTCGGAGGT TATTTATT	TCTATCACTCCCAGGT AGCTGCTCC
CD9	928	CGGCTTCCTCTTGG TGATATT	GCTTGTTGTAGGT GTCCTTGTA	ATGGCCGCAGCTATTT CAATGGC
SEMA7A	8482	TTCCGTACCTCCTC ACTCAA	TGGAAGGTCTCTG TGGGTAT	CTACCACTCAAGCCTT CCCAACCC
Jun	3725	CACAGAGAGACAG ACTTGAGAAC	ACTTGGATACCCT TGGCTTTAG	CTTGACAAGTTGCGAC GGAGAGAAA
JunB	3726	CCATCAACATGGA AGACCAAGA	CTTGAGCGTCTTC ACCTTGT	CATCAAAGTGGAGCG CAAGCGGC
RHOU	5848 0	GCAGGGAGGTGAA TACTCTTG	GAAGTACATCTTG GCCGACTTA	AAGGCCAACAGCAAG TGTTTGTGGGA
INSM1	3642	GTATGTAGCGTAC GGGTTGTT	CAGCAGTTCACAA GCCATAATAAA	AGGCTGTAAATTGAA CTTCCCACACGAT
RHOB	388	TTCGGAGCTAAGA TGGTGTTAT	GGAAAGATGGTC AAGTCCTGT	TAAGGGTGGTGATGG GTGAGCGC
EZR	7430	GATCGAGGATACT CACCAGAAA	CCAGTTGTATTTG GCTGGATTG	CACCATGGATGCAGA GCTGGAGTTT
LLGL2	3993	TGATCTCACCCTCG GAGTTT	GCGGTGGTTCTTG GTTTCT	CACTTGGTGGAGAGA GAGAAGCGC
SNX33	2573 64	CCGTAACCTCAACC GTTTCT	GGAGTATGTCTCA GCGATCTTG	ATGCTTTGTGCGTTCT GGAGTGGA
SPIRE2	8450 1	GTCTGCACTTCCTG TAGCATAA	GATACCCTCTGAG GACTCTCAA	CACATCCCTGTCTACA CACTGGGC
MLF1	4291	CTGGCTCCCGAGA	GCCTTTGATGTGG	AGGGAGAAACCTCAA

		ACTTAAA	AGTTTGTC	CAAAGTCCAGC
HSPA2	3306	CCCTGGAGTCCTAT ACCTACAA	GACACTTGTCGAG GATCTTGTT	AAGCAGACGGTGGAA GACGAGAAA
CDKN1A	1026	CGGAACAAGGAGT CAGACATT	AGTGCCAGGAAA GACAACTAC	AGATGGTGGCAGTAG AGGCTATGGA
ID2	3398	CAAGAAGGTGAGC AAGATGGA	GGTGATGCAGGCT GACAATA	TGCAGCACGTCATCGA CTACATCTTG
ERN1	2081	CCCATCAACCTCTC TTCTGTATC	AGGCCGCATAGTC AAAGTAG	TGGTGATGGTGTATTC TGTTCGCCC
NR2F2	7026	CGTCTCCTCCTCAG TCATAGA	TGCCATATACGGC CAGTTAAA	TGCTGCCGGACAGTA ACATATCCC
GAS2	2620	GATTGCAGCCAGG TATGGT	GAAGGAGAAGGT GAAGGAGAAG	TGGAGCCTCCTGGTTT GATAAAGCTG
CDK6	1021	CTAGCAACCATCCC TCCATTAC	CTCAGAGCATTCT GAAGACAGTAG	ACACAGAAAGCCCTC TTGAAGCAA
GAPDH	2597	GGTGTGAACCATG AGAAGTATGA	GAGTCCTTCCACG ATACCAAAG	AGATCATCAGCAATG CCTCCTGCA
UBC	7316	GTACCCTGTCTGAC TACAACATC	GTCTTGCCAGTGA GTGTCTT	AGAAAGAGTCCACCT TGCACCTGG

Table S3. Differential expressed genes between NNF- and INF-PAs.

This table is uploaded as excel file.

Table S4. The statistical significance of modules obtained from WGCNA.

**Modules**	**P**	**BH adjusted P**	**log10(adj. P)**
MEred	0.003	0.0072	2.1426675
MEblue	2.00E-05	0.00012	3.92081875
MEgreen	2.00E-04	0.0008	3.09691001
MEmagenta	0.005	0.01	-2
MEpink	0.7	0.7636364	0.11711338
MEblack	0.9	0.9	0.04575749
MEyellow	0.07	0.12	-0.9208188
MEgreenyellow	0.7	0.7636364	-0.1171134
MEpurple	0.1	0.15	-0.8239087
MEbrown	3.00E-04	0.0009	-3.0457575
MEturquoise	1.00E-05	0.00012	-3.9208188

Table S5. Clinical characteristics of NNF- and INF-PitNEt patients for validation.

**Clinicopathological feature**	**RT-PCR cohort**		*P* Value
	**NNF**	**INF**	
	92	51	

Age (years)			0.154 [a]
Average	52.8	46.4	
SD	14.2	14.0	
Gender			**0.007** [b]
Male	59	20	
Female	33	31	
P53			1.000 [c]
Negative	89	49	
Positive	3	2	
Ki-67			0.0869 [c]
≤3%	86	43	
＞3%	6	8	
Largest diameter			**0.001**[a]
average	28.6	36.4	
SD	7.4	12.6	

[a] Unpaired t test; [b] Chi-square test; [c] Fisher's exact test.

Boldface type indicates statistical significance.

Table S6. Extent of INSM1 staining in PA subtypes.

Subtypes	Invasion	INSM1 moderate & strong / positive (percentage)	Percentage of positive tumor cell nuclei				P value (H-score)
			<40%	≥40 and <60%	≥60 and <80%	≥80	
SGA	NNF-PA	15/40 (37.50%)	8	5	20	7	0.4085
	INF-PA	6/8 (75%)	1	1	2	2	
Nul cell adenomas	NNF-PA	2/8 (25%)	2	2	2	2	***0.0497***
	INF-PA	3/5 (60%)	0	0	3	2	
SCA	NNF-PA	5/5 (100%)	0	0	0	5	0.8651
	INF-PA	13/14 (92.86%)	0	0	3	11	

Table S7. Extent of HSPA2 staining in PA subtypes.

Subtypes	Invasion	HSPA2 positive / total (percentage)	Percentage of positive tumor cells				P value (H-score)
			<40%	≥40 and <60%	≥60 and <80%	≥80	
SGA	NNF-PA	17/39 (43.59%)	4	3	8	2	0.7532
	INF-PA	5/8 (62.50%)	2	2	1	0	
Null cell adenomas	NNF-PA	4/10 (40%)	0	0	2	2	0.8228
	INF-PA	3/5 (60%)	0	1	2	0	
SCA	NNF-PA	3/5 (60%)	0	0	1	2	0.7374
	INF-PA	14/14 (100%)	3	1	5	5	

Table S8. Extent of CDK6 staining in PA subtypes.

Subtypes	Invasion	CDK6 positive / total (percentage)	Percentage of positive tumor cells				P value (H-score)
			<40%	≥40 and <60%	≥60 and <80%	≥80	
SGA	NNF-PA	34/42 (80.95%)	11	6	14	3	***0.0435***
	INF-PA	4/6 (66.67%)	3	1	0	0	
Null cell adenomas	NNF-PA	6/9 (66.67%)	3	0	1	2	0.1528
	INF-PA	5/5 (100%)	1	2	1	1	
SCA	NNF-PA	4/5 (80%)	2	2	0	0	0.0684
	INF-PA	14/15 (93.33%)	2	2	8	2	
